# The *In Vivo* Transcriptomic Blueprint of *Mycobacterium tuberculosis* in the Lung

**DOI:** 10.3389/fimmu.2021.763364

**Published:** 2021-12-22

**Authors:** Mariateresa Coppola, Rachel P-J. Lai, Robert J. Wilkinson, Tom H. M. Ottenhoff

**Affiliations:** ^1^ Department Infectious Diseases, Leiden University Medical Center (LUMC), Leiden, Netherlands; ^2^ The Francis Crick Institute, London, United Kingdom; ^3^ Department of Infectious Diseases, Imperial College London, London, United Kingdom; ^4^ Department of Medicine, Institute of Infectious Disease and Molecular Medicine, Wellcome Centre for Infectious Diseases Research in Africa, Cape Town, South Africa

**Keywords:** Mycobacterium tuberculosis (MTB), transcriptomic, tuberculosis, vaccine, therapy, antigen discovery

## Abstract

*Mycobacterium tuberculosis* (*Mtb*) genes encoding proteins targeted by vaccines and drugs should be expressed in the lung, the main organ affected by *Mtb*, for these to be effective. However, the pulmonary expression of most *Mtb* genes and their proteins remains poorly characterized. The aim of this study is to fill this knowledge gap. We analyzed large scale transcriptomic datasets from specimens of *Mtb*-infected humans, TB-hypersusceptible (C3H/FeJ) and TB-resistant (C57BL/6J) mice and compared data to *in vitro* cultured *Mtb* gene-expression profiles. Results revealed high concordance in the most abundantly *in vivo* expressed genes between pulmonary *Mtb* transcriptomes from different datasets and different species. As expected, this contrasted with a lower correlation found with the highest expressed *Mtb* genes from *in vitro* datasets. Among the most consistently and highly *in vivo* expressed genes, 35 have not yet been explored as targets for vaccination or treatment. More than half of these genes are involved in protein synthesis or metabolic pathways. This first lung-oriented multi-study analysis of the *in vivo* expressed *Mtb*-transcriptome provides essential data that considerably increase our understanding of pulmonary TB infection biology, and identifies novel molecules for target-based TB-vaccine and drug development.

## Introduction

Tuberculosis (TB) is an ancient disease caused by the airborne pathogen *Mycobacterium tuberculosis* (*Mtb*), which has infected billions of people and has killed more people than any other bacterial infectious agent (1). Current tools to combat TB, including diagnostics, vaccines and drugs, each for their own reasons, are insufficient to diminish the global TB burden. In response to this WHO has formulated consensus research priorities to accelerate the discovery and development of better TB drugs, diagnostics and vaccines (1, 2).

The current clinical TB vaccine pipeline includes more than 20 new candidates, which in pre-clinical animal models are able to reduce *Mtb* infection in the lung (quantified by measuring numbers of viable *Mtb* and/or pathology in the lung) and/or systemically (often the spleen), or TB morbidity (quantified by tissue pathology, weight loss or host survival) (1, 3). Recent human phase II studies showed that sustained *Mtb* infection can be prevented by BCG revaccination of *Mtb* uninfected individuals (as measured by interferon gamma release assay conversion), and that incidence of new TB cases can be reduced amongst persons with LTBI by the M72 TB subunit vaccine (4, 5). While these results are highly encouraging, the immune mechanisms underlying these protective effects remain poorly understood. Current understanding of the molecular and cellular basis of *Mtb*/human host interaction is limited, and the exact mechanisms exerting protection against TB remain largely unknown, and could range from (trained) innate to adaptive immune effector mechanisms (6). Many of the most advanced TB subunit candidates are based on *Mtb* proteins historically identified from *in vitro* cultured *Mtb* which were thereafter tested for antigenicity using peripheral blood cells from *Mtb* exposed individuals (7).

Although immune recognition of such proteins suggests previous immune priming by these antigens, it does not provide information regarding the expression of these antigens in the main organ affected by *Mtb*, the lung. We have hypothesized that in order for vaccine antigens to be protective (and for drug targets to be effective), a minimal requirement is that they are expressed in the lung of *Mtb* infected individuals, preferably over prolonged periods of time such that immune cells can recognize and eliminate infected targets cells presenting these antigens; or conversely, that TB drugs are able to act effectively on such *Mtb* target molecules. To address this important issue, which cannot easily be studied in humans, we recently reported a novel *in vivo* pulmonary *Mtb* gene expression analysis based on the *Mtb* RNA expression patterns in the lung of highly susceptible (C3HeB/FeJ) as well as resistant (C57BL/6J) mice after aerosol *Mtb* (Erdman) infection, from early to late time points (8). Of note, the highly susceptible C3HeB/FeJ “Kramnik” mice uniquely develop a form of lung TB that includes centrally necrotic lesions characteristic of human TB. By analysing the expression of 2068 *Mtb* genes [selected to represent the first genes of most predicted *Mtb* operons, in order to enhance representative “genome wide” coverage (9)] in their lungs during early and late phase *Mtb* infection, we identified upregulated *Mtb* genes that were designated *in vivo* expressed *Mtb* (IVE-TB) genes. A total of 50 IVE-TB candidate antigens was selected further based on information including high-level conservation among whole-genome sequenced *Mtb*-complex strains (n = 219) and algorithms predicting epitopes presented by HLA-class-Ia and -II. Many of the IVE-TB candidate antigens were highly conserved among the genomes of >200 human isolated *Mtb* strains, and were recognized by *Mtb* responsive human subjects, supporting the hypothesis that the IVE-TB genes found in the C3HeB/FeJ “Kramnik” mouse lung were also expressed and presented in humans, likely in the lung.

Nevertheless, formal proof that these *Mtb* IVE-TB genes are truly expressed in the *Mtb* infected human lung is lacking. Only few studies have evaluated the *Mtb* transcriptome by human lung infection centred approaches, using sputum and bronchoalveolar lavage (BAL) samples from pulmonary TB patients. Those few studies used mostly quantitative real-time PCR (RT-PCR) or microarray platforms (10–15). In these reports, the expression levels of *Mtb* genes recovered from human sputum were compared to those in *in vitro* cultures. Up- and down-regulated *Mtb* genes were reported mainly as ‘fold-changes’ compared to *in vitro* cultured *Mtb* or evaluated as quantitatively altered *Mtb* gene expression profiles in samples collected during TB treatment. Since a direct comparison between these ‘fold-changes’ and quantitative qPCR data was not immediately possible, we collected and reanalyzed all raw data and relative expression scores of *Mtb* transcripts from three studies (11, 12, 15). These human transcriptomic data sets were selected because raw data were publicly available and allowed comparative analysis. Ranking *Mtb* genes within each dataset allowed us to assess and combine *in vivo Mtb* gene expression in multiple individual studies independent of their individual design. Using this information and comparing three independent *Mtb* transcriptome datasets from human sputum or BAL with our previously published dataset from C3HeB/FeJ “Kramnik” mouse lungs (8), which was then complemented by validation in a recently deposited *Mtb* RNA-Seq dataset (GEO: GSE132354) (16) from alveolar macrophages of *Mtb* infected C57BL/6J mice, as well a partial validation in a *Mtb* RNA-Seq dataset from seven human active TB sputum samples (GEO: GSE137518) (17), we here provide a first lung-centric multi-study transcriptomic integrated dataset, which provides a novel tool likely to be useful to TB vaccine and drug target discovery.

## Results

### Most Abundant *Mtb* Transcripts in Human Airway Overlap With Those in *Mtb*-Hypersusceptible-Mice Lung

Three published quantitative RT-PCR datasets were used to assess the most abundant *Mtb* transcripts across host species [mice (n=20) (8) and humans (n=28) (11, 12)] and lung-derived specimen-type [murine lungs (n=20) (8), human BAL (n=11) (12) and sputum (n=28) (11, 12)] (a brief description of the datasets is provided in **Table 1**). As mentioned above, the number of *Mtb* genes examined in these studies ranged between 1970 and 2406, mostly selected to represent the first genes of predicted *Mtb* operons.

**Table 1:** Description of the *Mtb* transcriptomic datasets included in the study.

Dataset	Host	Type of Sample	Number of Samples	Sample characteristics	Method	Number of genes analysed	Data availability	Reference
**a**	C3HeB/FeJ (C3H) mice	Lung tissue	20	2, 4, 6, 9 and 12 weeks post *Mtb* Erdman aerosol challenge	RT-PCR	2068	**Data File S1.1**	Coppola M, et al., 2016 (8)
**b**	TB patients recruited in Uganda	Sputum	17	Before TB treatment	RT-PCR	2406	**Data File S1.2**	Walter ND, et al., 2015 (11)
**c1**	TB patients recruited in South Africa	Sputum	11	Before TB treatment	RT-PCR	1970	**Data File S1.3**	Garcia BJ, et al., 2016 (12)
**c2**	TB patients recruited in South Africa	BAL	11	Before TB treatment	RT-PCR	1970	**Data File S1.3**	Garcia BJ, et al., 2016 (12)
**c3**	–	*in vitro* culture	4	H37Rv cultured in log phase aerobic growth	RT-PCR		**Data File S1.4**	Garcia BJ, et al., 2016 (12)
**c4**	–	*in vitro* culture	6	H37Rv cultured in non-replicating persistence state (NRP-2)	RT-PCR		**Data File S1.4**	Garcia BJ, et al., 2016 (12)
**d**	TB patients recruited in India	Sputum	7	Before TB treatment	Microarray	3924	**Data File S1.5**	Sharma S, et al., 2017 (15)
**e**	C57BL/6J mice	Alveolar macrophages isolated from lungs	3	2 weeks post *Mtb* smyc’::mCherry Erdman challenge	RNA-Seq	3766	**Data File S1.6**	Pisu D, et al., 2020 (16)
**f**	TB patients recruited in South Africa	Sputum	7*	Before TB treatment	RNA-Seq	Range: 2957-3993	**Data File S1.7**	Lai R, et al., 2021 (GSE137518) (17)

Mtb, Mycobacterium tuberculosis; TB, Tuberculosis; BAL, bronchoalveolar lavage; RT-PCR, quantitative real-time PCR; RNA-Seq, RNA-Sequencing. *one is a TB-HIV patient.

**Figure 1:**
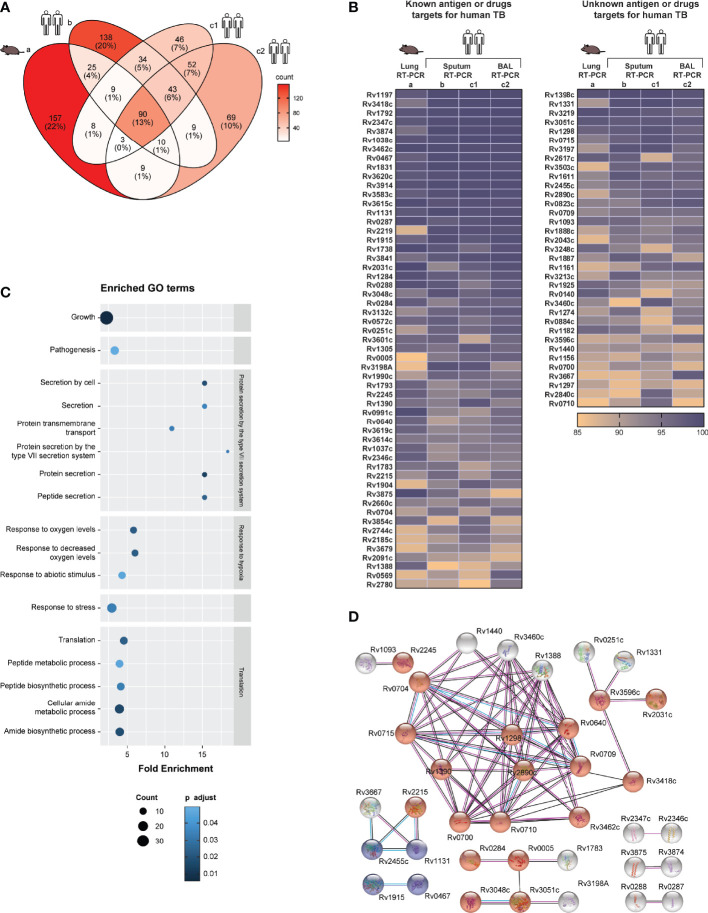
Most abundant *Mtb* transcripts in human airway overlap with those in *Mtb*-hypersusceptible-mice lung. **(A)** Venn diagram indicating the numbers of top 15% expressed *Mtb* genes that overlap among four published *in vivo* RT-PCR datasets from the lungs of infected C3HeB/FeJ (C3H) mice (8) (dataset a), human sputa (11, 12) (datasets b and c1) and human BAL samples (12) (dataset c2). **(B)** The top ranked expressed *Mtb* genes (n=90) overlapping among all these *in vivo* datasets, see **(A)**, are shown in two heatmaps. The left heatmap lists genes that have been investigated as antigens or TB drugs; the right heatmap lists genes of which the vaccine or drug potential remains unexplored. The color coding represents the expression rank for a certain gene (rows) in each dataset (columns). The genes, identified by their Rv numbers, are ordered based on expression rank from top to bottom. **(C)** GO enrichment analysis performed on the top expressed *Mtb* genes (n=90), see **(A, B)**. The fold enrichments, the adjusted p value (Fisher, FDR) and the number of genes within each GO term, are shown only for biological processes significantly enriched. **(D)** STRING network analysis performed on the top expressed *Mtb* genes (n=90), see **(A, B)**. Blue nodes indicate biological process in tricarboxylic acid metabolic process and red nodes indicate growth. The color of the lines indicates the following: light blue = known interaction from curated databases; pink = known interaction from experiments; red = predicted gene fusions; blue = predicted gene co-occurrence; black = co-expression. Disconnected nodes were hidden.

Before comparison, within each dataset a relative score from 0 to 100 was calculated by scaling the expression values of all detected *Mtb* genes, with 0 and 100 representing the lowest and highest expressed genes, respectively (**Data File S1**: **Tables S1.1, S1.2, S1.3**). By comparing the most abundant *Mtb* transcripts, arbitrarily defined as genes having a relative expression rank of 85% or higher, 90 *Mtb* genes were identified as overlapping in this cross-study analysis (**Figure 1A**). Interestingly, 49% of the top expressed *Mtb* genes in the *Mtb*-hypersusceptible-mice lung overlaps with those found in human sputum or human BAL (**Figure 1A**). If considering the human data only, the top ranked genes would mount up to 133 overlaps (**Data File S1**: **Table S1.8**). However, since mice are the most commonly used model for preclinical research on TB vaccine and drug development, we will focus the following analysis on the most abundant *Mtb* transcripts shared between humans and mice.

To validate these 90 *Mtb* transcripts found to be consistently highly expressed in different human and mouse lung-derived TB datasets (**Figure 1B**), we compared these findings to unbiased *Mtb* RNA-Sequencing (RNA-Seq) data from alveolar macrophages of *Mtb* infected C57BL/6J mice (**Data File S1**: **Table S1.6**, dataset e) (GSE132354) (**Table 1**). Out of these 90 *Mtb* genes, 80 were also listed in the mouse lung-derived RNA-Seq library and except for three genes (Rv1037c, Rv1038c and Rv3619c) they were detectable in all samples studied (i.e., more than five reads). In addition, among the top 15% expressed *Mtb* genes detected in the mouse RNA-Seq dataset (104 out of 3766, **Data File S1**: **Table S1.6**), 17 (Rv0005, Rv0284, Rv1161, Rv1297, Rv1398c, Rv1611, Rv1783, Rv1925, Rv2031c, Rv3051c, Rv3219, Rv3248c, Rv3583c, Rv3841, Rv3854c, Rv3874, and Rv3875) were shared with the RT-PCR datasets. Furthermore, a partial validation was performed by using a *Mtb* RNA-Seq dataset from seven human active TB sputum samples (**Data File S1**: **Table S1.7**, dataset f) (GSE137518) (17) (**Table 1**): 79 out of 90 *Mtb* antigens were detectable in all samples, seven (Rv1131, Rv1738, Rv1888c, Rv2031c, Rv3108A, Rv0569 and Rv0991c) in 6 samples, three (Rv0572c, Rv1037c and Rv3619c) in 5 samples and one (Rv2660c) in 3 samples. In addition, among the top 15% expressed *Mtb* genes detected in the human RNA-Seq dataset (24 out of 3716, **Data File S1**: **Table S1.7**), eight (Rv3874, Rv0467, Rv1398c, Rv1131, Rv3051c, Rv0284, Rv3248c and Rv1297) were shared with the RT-PCR datasets.

We also performed a similar analysis on a published microarray *Mtb* transcriptome (number of genes=3924) obtained from human sputum samples (n=7) (15) (**Data File S1**: **Table S1.5**) (**Table 1**). Of the most abundant 90 *Mtb* transcripts found in the RT-PCR datasets, only seven genes (Rv1915, Rv1388, Rv3667, Rv0715, Rv1738, Rv3601c, and Rv0287) overlapped with the top 15% ranked genes of the published microarray dataset. Microarray data are known to be less quantitative and to have a lower dynamic range compared to RT-qPCR, which probably underlies this relatively limited correlation (18).

Strikingly, 51 of the most abundant transcripts have previously been described as *Mtb* antigens and four (Rv0005, Rv1305, Rv3601c, and Rv3854c) as targets of approved TB drugs (7, 19, 20) (**Figure 1B**, left panel). To the best of our knowledge, 35 of the top ranked *Mtb* transcripts have not been examined yet as potential targets for TB vaccination or treatment (7, 19, 20) (**Figure 1B**, right panel). Among this pool of unexplored genes, the majority encode ribosomal proteins or proteins needed for energy-transducing processes, thus essential for *Mtb* growth and survival.

Among the above identified 90 abundantly expressed *Mtb* “pulmonary core *Mtb* transcript set”, significant enrichment was observed in genes related to specific biological processes (21) including: *Mtb* growth, pathogenesis, response to hypoxia, response to stress, translation, and protein secretion by the type VII secretion system. The latter process was the most enriched, compatible with its major role in virulence and pathogenesis (**Figure 1C**). By using STRING, most of the predicted protein-protein interactions were found among those involved in growth (especially ribosomal proteins), tricarboxylic acid metabolic process, and secreted/virulent proteins (**Figure 1D**). Furthermore, multiple *Mtb* genes among the 90 consistently highly expressed have previously been associated with key functional categories (11, 22). These include: genes induced by oxidative, non-specific and stringent stress as well as by enduring hypoxia (EHR); regulatory genes, and genes involved in aerobic or anaerobic respiration. Furthermore, several *esx* genes were highly expressed in all datasets (**Figure S1**). Genes encoding for phage proteins, toxin and antitoxins, and for PE/PPE proteins were often not determined in RT-PCR datasets, and were detected at low levels in the other datasets (**Figure S1**). Similarly, many genes associated with cholesterol metabolism and its regulon were not determined or not highly expressed in the RT-PCR datasets, with the exception of Rv3503c (**Figure S1**) (22).

Taken together, this first cross-study analysis identified a pulmonary core *Mtb* transcript set of 90 *Mtb* genes that were highly and consistently expressed among different datasets analyzed by qRT-PCR. Of note, the detection of most of these genes was validated in mouse as well as human lung-derived RNA-Seq datasets. Importantly these genes encode for proteins involved in biological processes essential to survival and likely also transmission of *Mtb*. Interestingly, the vaccine or drug potential of 35 top ranked genes remains unexplored. When further confirmed, including at the proteomic level, these results significantly increase our understanding of *in vivo Mtb*-infection biology, and provide novel targets for innovative TB vaccine and drug discovery.

### *In Vivo* and *In Vitro Mtb* Transcriptome Dataset Comparison

**Figure 2:**
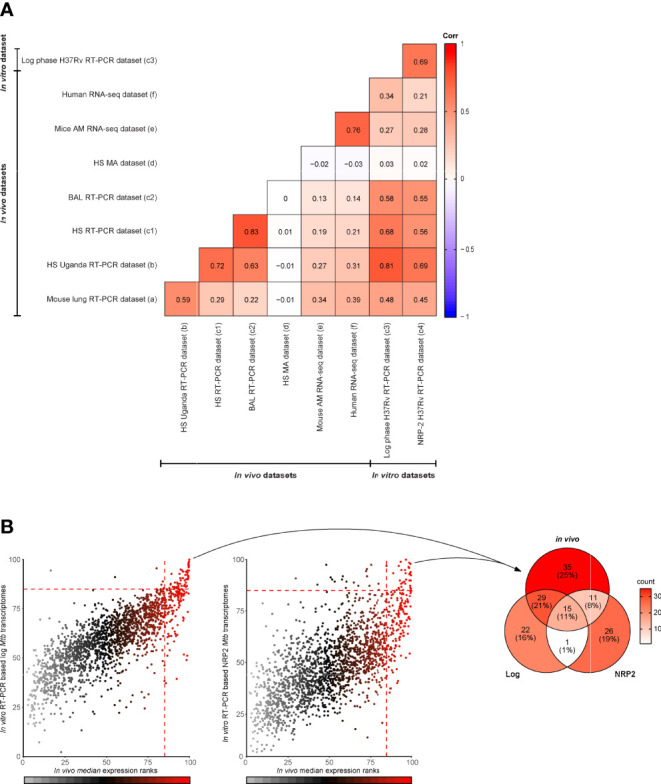
*In vivo* and *in vitro Mtb* transcriptome dataset comparison. **(A)** Correlation between *in vivo* and *in vitro Mtb* transcriptome datasets (n=nine) measured using Spearmans rank correlation coefficients “r” (from 1 to -1). This comparison was based only on the ranked expression of *Mtb* genes (n=1813) investigated in all nine *Mtb* transcriptomes. The datasets included are the following: C3HeB/FeJ mouse lung dataset (8) (a); human sputum (HS) dataset from Ugandan TB patients (10) (b); HS (c1) and BAL (c2) from a cohort of South African TB patients (12); a microarray (MA) based dataset (15) (d) from sputa of Indian TB patients (15); RNA-Seq dataset from alveolar macrophages (AM) of *Mtb* infected C57BL/6J mice (GEO: GSE132354)] (e); and RT-PCR based datasets from log phase aerobic grown *Mtb* H37Rv (c3) and non-replicating persistence state (NRP-2) cultured *Mtb* H37Rv (c4). **(B)** Scatter plots (left and middle panel) displaying the relationship between the median expression ranks of the four published *in vivo* RT-PCR based *Mtb* transcriptomes (datasets a, b, c1 and c2) (x-axes) and the median expression ranks of the two published *in vitro* RT-PCR based *Mtb* transcriptomes (c3 and c4, left and right plot, respectively) (y-axes). Dots are color coded according to the median expression ranks of the *in vivo* datasets. At the value of 85%, dotted lines intersect the axes indicating the top 15% expressed *Mtb* genes. Venn diagram (right panel) showing the numbers of top ranked *in vivo Mtb* genes (n=90), described in **Figure 1**, that overlap with the top ranked *in vitro Mtb* genes from log phase aerobic grown *Mtb* H37Rv and non-replicating persistence state (NRP-2) cultured *Mtb* H37Rv.

Although there was high concordance between qRT-PCR datasets from the different studies, as expected there also were some differences in the most abundantly expressed genes between qRT-PCR, microarray and RNA-Seq datasets. To compare all nine *Mtb* transcriptome datasets analyzed in this study we calculated Spearmans rank correlation coefficients “r” among each of the datasets. In this analysis we also examined two additional *in vitro Mtb* RT-PCR transcriptome datasets (12) (a brief description of the datasets is provided in **Table 1**). The entire datasets-comparison consisted of the ranked expression of *Mtb* genes (n=1813) that were commonly investigated in all nine *Mtb* transcriptomes. Most of the *in vivo* datasets correlated positively (**Figure 2A**). The strongest *in vivo* data correlation (r=0·83) was found within *Mtb* transcriptome datasets based on RNA obtained from BAL and sputum from the same individuals (South Africa cohort), and results obtained with the same technology platform (RT-PCR) as previously described in the original manuscript (12). The RT-PCR dataset based on human sputum of Ugandan TB correlated most strongly with other datasets including those from the South Africa TB patient cohort (sputum and BAL), the RNA-seq dataset from the seven South Africa TB patients’ sputa and with both the RNA-Seq and the RT-PCR datasets from *Mtb* infected mice (r values were 0·72, 0·63, 0·31, 0·27, and 0·59, respectively). Of all the *in vivo* datasets the latter showed the highest correlation with the mouse and human RNA-Seq transcriptome datasets (r values were 0·34, and 0·39). Importantly, the mouse RNA-Seq transcriptome dataset highly correlated with the RNA-Seq data derived from human samples (r=0·76).

The microarray *Mtb* transcriptome dataset did not correlate well with any of the other datasets investigated here, probably for the reasons described above (see also **Figure 2A**). When comparing the *in vivo* and *in vitro Mtb* transcriptome based on the RT-PCR datasets, the expression ranks of the *in vivo* data correlated most strongly with the expression ranks obtained *in vitro* from log phase grown *Mtb* (datasets c3) (**Figure 2A**). However, despite the overall positive correlation, the top ranked expressed genes identified under the *in vitro* conditions.

Only partially overlapped with the *in vivo* pulmonary core *Mtb* transcript set identified in the *in vivo* datasets, strongly suggesting different biology (**Figure 2B**). Confirming what already was described for the whole datasets, the overlaps were higher with the top ranked genes from log phase aerobic grown *Mtb* H37Rv (n=44) than those from the non-replicating persistence state cultured *Mtb* H37Rv (n=26) (**Figure 2B**) (**Data File S1**: **Table S1.9**). Of note, the overlaps between the top ranked genes of the two different experimental models were lower than those found when comparing the *in vitro* datasets individually to the *in vivo* transcriptomes (n=16).

Thus, this latter comparison showed that the expression ranks of *Mtb* genes identified in *in vivo* and *in vitro* positively correlated among multiple datasets, including those derived from different species. In addition, it also revealed that the set of highly *in vivo* expressed *Mtb* genes substantially differs from the set of highly *in vitro* expressed *Mtb* genes.

### Expression of *Mtb* Genes Encoding Candidate Antigens for TB Vaccines and Diagnostics Across Datasets

**Figure 3:**
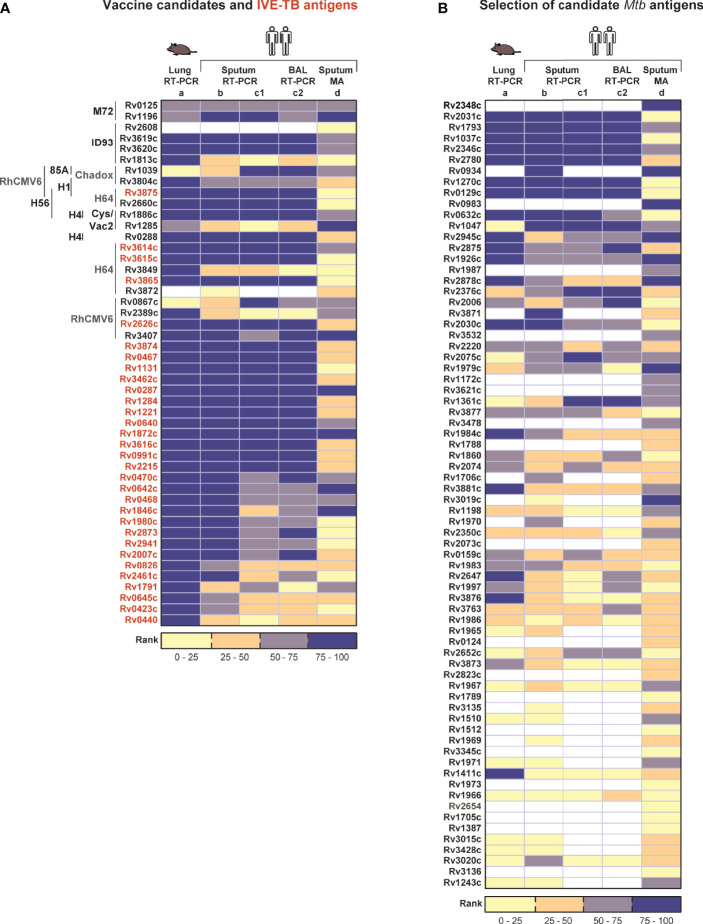
Expression of *Mtb* genes encoding candidate TB vaccine antigens and IVE-TB antigens across datasets. The median expression level of *Mtb* genes encoding a selection of the most promising *Mtb* antigens previously identified was interrogated from published (8, 11, 12, 15) lung *Mtb* infection-centred transcriptomic datasets. Each column represents the relative expression rank calculated obtained from our analysis within each dataset, as indicated in the boxes by numbers. Source data are provided in **Supplementary Table 1** **(A)** Proteins constituting clinical or late preclinical subunit TB vaccine candidates (n=22) as well as IVE-TB antigens (font color red, n=30) previously identified. On the left of each antigen is indicated the name of the vaccine candidates of which they are part (font colors in black or grey differentiate candidates in clinical and pre-clinical studies, respectively). **(B)** Expression ranks of genes used in commercial diagnostics (Rv2348c, font color grey) and genes encoding *Mtb* proteins described as promising antigens (n=70) by at least two previous independent studies other than the IVE-TB approach (7, 23). Undetermined expression ranks are color coded in white. Datasets are listed from left to right as in Figure 2 (see legend **Figure 2**).

In order to be effective, TB vaccines would need to contain antigens consistently expressed by *Mtb* at the site of infection, the lung, and across various diverse populations. We therefore interrogated published (8, 11, 12, 15) *Mtb* transcriptome datasets to evaluate the expression levels of genes encoding proteins constituting subunit TB vaccine candidates (n=22) (7) as well as a selection of the most promising IVE-TB antigens previously identified (n=30) (8). As described above, to compare multiple datasets we used normalized and ranked *Mtb* gene expression values. Two genes, namely Rv2608 and Rv3872 belonging to the PE/PPE family, were not determined in all RT-PCR datasets. As expected from the previously described general comparison (**Figure 2A**), the microarray dataset showed the most discordant expression levels with the exception of a few genes (**Figures 3A, B**). Next, *Mtb* genes were assigned to the first, second or third quartiles based on the median expression rank across the qRT-PCR and microarray datasets. Among 22 *Mtb* genes encoding current TB vaccine candidates, 12 ranked in the upper quartile (Rv3615c, Rv3620c, Rv0288, Rv3614c, Rv3619c, Rv3875, Rv2660c, Rv2626c, Rv3865, Rv1196, Rv3407, and Rv1886c), nine ranked in the interquartile range (Rv0867c, Rv3804c, Rv0125, Rv0139, Rv1285, Rv2389c, Rv3849, and Rv1813c) and only two ranked in the lower quartile (Rv2608, and Rv3872) (**Figure 3A**). By performing the same analysis, among the most promising 30 IVE-TB genes, 20 ranked in the upper quartile (Rv0467, Rv3462c, Rv1131, Rv3615c, Rv0287, Rv0288, Rv1284, Rv1221, Rv1872c, Rv0640, Rv3614c, Rv3616c, Rv0991c, Rv2215, Rv2626c, Rv0470c, Rv0642c, Rv0468, Rv3865, and Rv1846) and ten ranked in the interquartile range (Rv1980c, Rv2873, Rv2941, Rv2007c, Rv0826, Rv2461c, Rv1791c, Rv0645c, Rv0423c, and Rv0440) (**Figure 3A**). All these genes, except nine (detected in six samples: Rv0826, Rv0991, Rv2873, Rv3407; detected in five samples: Rv1285, Rv2608, Rv2626, Rv3619; detected in three samples: Rv2660c), were detected in all human samples analyzed by RNA-Seq (dataset f) whereas 44 were traceable in the mouse RNA-Seq dataset (dataset e) and among those only one (Rv3619) was not detected (i.e., less than five reads) in the mouse samples analyzed by that platform. Among genes used in commercial immune- (interferon gamma release assay, IGRAs) diagnostics, we found the expression of ESAT-6 (Rv3875c) and CFP-10 (Rv3874) to rank in the upper quartile (**Figure 3A**), and TB7.7 (Rv2654) in the lower quartile (**Figure 3B**). All the genes encoding for antigens proposed in an ESAT-6 free immunodiagnostic test (Rv2348c, Rv3615c, and Rv3865) were expressed among the upper quartile (23) (**Figures 3A, B**).

As mentioned above, multiple *Mtb* antigens have been discovered over the last 40 years using different strategies other than our IVE-TB approach. For our expression analysis here, we focused on 70 genes encoding *Mtb* proteins which have been described as promising antigens and validated in at least two independent studies (7). Of the 70 *Mtb* genes only 11 had expression median ranks in the upper quartile (Rv2031c, Rv1793, Rv2346c, Rv1037c, Rv2780, Rv0934, Rv0983, Rv0129c, Rv1270c, Rv0632c, and Rv1047) and were detected in all samples by RNA-Seq (with the exception of Rv1037 and Rv1047) (**Figure 3B**). Finally, we performed a similar analysis on 89 *Mtb* genes encoding peptides that have been included in the so-called mega-pool of epitopes which has been widely tested in numerous different settings (24, 25). Out of 89 *Mtb* genes, 29 had an expression rank in the upper quartile (**Figure S1**). Among these highly *in vivo* expressed genes 12 (Rv3418c, Rv3023c, Rv1199c, Rv0985c, Rv3115, Rv3022c, Rv2512c, Rv0299, Rv0294, Rv3018c, Rv2996c, and Rv1908c) were not previously described as IVE-TB genes or implemented in TB vaccines or diagnostics (**Figure S1**).

Taken together, these analyses show that most potent candidate TB vaccine antigens, some of which are in advanced clinical evaluation studies, are indeed highly expressed at the RNA level in infected lungs of mice and humans. This supports our previous hypothesis that *Mtb* antigen expression in the lung (if confirmed at the protein level) is a critical feature of potential vaccine efficacy, and that this could guide target selection to advance and improve TB vaccine development as well as new TB diagnostics.

### Expression Profiles of *Mtb* Genes Encoding TB Drugs Targets

**Figure 4:**
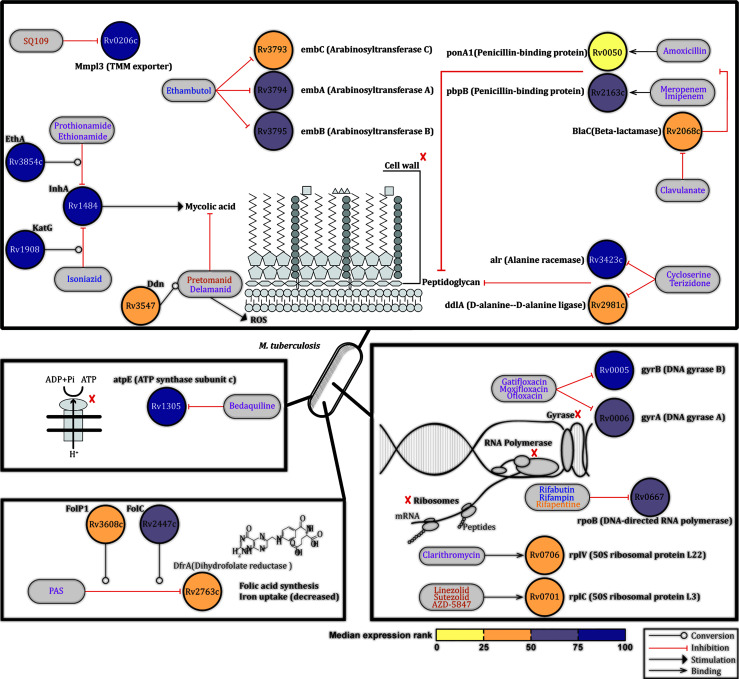
Expression profiles of *Mtb* genes encoding TB drugs targets. Based on the information available on the TBDRUGS - Database of Drugs for Tuberculosis (version 1.0), available at http://bmi.icmr.org.in/tbdrugs/, the median expression ranks of 22 *Mtb* genes encoding TB drugs targets or prodrug-drug converting enzyme were evaluated across published (8, 10, 11, 14) lung *Mtb* infection-centred transcriptomic datasets. Nodes representing genes are colored based on the median expression rank. Blue, magenta and orange text indicate first-line drugs, other approved drugs or drugs evaluated in clinical trials, respectively. Source data are provided in **Supplementary Table 1**.

Twenty-eight TB drugs have been approved for use against TB (20). Since these drugs can cure drug sensitive TB, we would predict that their targets, which are mostly proteins, should be expressed particularly during pulmonary *Mtb* infection. Based on the information available on the TBDRUGS - Database of Drugs for Tuberculosis (version 1.0) at http://bmi.icmr.org.in/tbdrugs/, we evaluated the median expression ranks across four published studies of 22 *Mtb* genes encoding major TB drugs targets, or prodrug-drug converting enzymes, across the qRT-PCR, and microarray transcriptomic datasets (**Figure S1**). Similar to the above, *Mtb* genes were again assigned to the first, second and third quartiles based on the median expression rank across the qRT-PCR and microarray datasets. Seven *Mtb* genes which ranked in the upper quartile (Rv1305, Rv0005, Rv3854c, Rv3423c, Rv1484, Rv1908c, and Rv0206c) are targets (or prodrug-drug converting enzymes) of drugs inhibiting the synthesis of mycolic acids (Isoniazid, Prothionamide and Ethionamide), peptidoglycans (Cycloserine and Terizidone), or DNA (Gatifloxacin, Moxifloxacin and Ofloxicin), or interfering with trehalose monomycolate export (SQ109), or interfering with oxidative phosphorylation (Bedaquiline) (**Figure 4**). All other genes encoding drug targets had median expression ranks in the interquartile range (Rv0667, Rv3794, Rv3795, Rv2447, Rv2163c, Rv0006, Rv3608, Rv3547, Rv2068c, Rv0706, Rv0701, Rv3793, and Rv2981c), with the exception of the target gene (Rv2763c) of the pro-drug Para-aminosalicylic acid (PAS) which had an expression rank in the lower quartile (**Figure 4**). Of note, all 22 *Mtb* genes reviewed here, except three (Rv3608 and Rv2447 not traceable in the mouse dataset, Rv3608 and Rv2763c not found in some of the human samples), were detected in all samples evaluated by RNA-Seq analysis (**Data File S1**: **Tables S1.6, S1.7**).

Thus the 22 *Mtb* genes evaluated here which encode targets for drugs efficacious clinical TB-drugs are all expressed by *Mtb* in the mouse and human lung across multiple datasets.

Taken together, our analyses significantly increase current knowledge on the *in vivo* expression of the pulmonary *Mtb* transcriptome expression. These insights and new data could be highly valuable in guiding rationalized target selection of new TB antigens (for both diagnostics and vaccines) as well as TB drug discovery.

## Discussion

This study represents the first lung-oriented description of *Mtb* transcripts from sputum and bronchoalveolar lavage (BAL) of TB patients and from the lungs of *Mtb* infected mice (8, 11, 12, 15). Our analysis identified considerable overlap among the most abundant *Mtb* transcripts in different settings, spanning information from mouse (C3H/FeJ) and human *Mtb* infection. The only exception were microarray based data, probably due to the lower dynamic range of that platform (discussed further below). These findings were further validated and corroborated using mouse and human sputum *Mtb* transcriptomes measured by RNA-Seq. In addition, the data were compared with *in vitro* datasets, revealing significant differences as expected.

An important observation was that many *Mtb* genes encoding new candidate *Mtb* vaccine antigens or existing drug targets were among the most highly expressed *Mtb* genes in the lungs of infected mice and humans. A second important observation was that our analysis revealed high expression of 35 *Mtb* genes that have not been studied as vaccine antigens, diagnostic or drug target molecules, suggesting that the here reported data might inform novel strategies for discovery of new antigens and drug targets. Thus, this first comprehensive lung infection centred *Mtb* gene expression comparison provides novel insights into the pulmonary *Mtb* transcriptome signature and the biological pathways involved, significantly enhances our understanding of *Mtb* infection biology and is useful in deciphering the *in vivo* expressed pulmonary *Mtb* transcriptome, which can guide precision development of new TB diagnostics, drugs and vaccines (7).

Assessing the *Mtb* transcriptome in infected human organs is challenging since airway specimens such as sputum, BAL and biopsies are not always easy to obtain. Among those, sputum collection is the only non-invasive procedure and therefore the most commonly used to investigate *Mtb* transcriptomes in TB (10–15). However, adequate RNA isolation from sputum is hampered by several factors such as the small size of the samples, the presence of RNases, and the contamination with other commensal or pathogenic bacteria (26). In past studies, we circumvented those limitations by analysing RNA isolated from lung of *Mtb* infected mice, including those of highly susceptible C3HeB/FeJ “Kramnik” mice presenting with pulmonary lesions characteristic of human TB (8, 27), using RT-PCR. Although these methods allowed the identification of a new class of *in vivo expressed Mtb* (IVE-TB) antigens, proven to be recognized by peripheral blood cells of *Mtb* infected individuals (8, 27), it was not clear whether the *Mtb* transcriptome signature found in the lung of “Kramnik” mice sufficiently reflected that of clinical *Mtb* isolates infecting the human respiratory tract. The present comparison shows a strongly positive correlation between *Mtb* transcriptomes signatures from infected lungs of “Kramnik” mice and three out of four published *Mtb* transcriptome signatures identified from sputum and BAL of TB patients (**Figure 2**). The only *Mtb* transcriptome dataset that did not correlate with the mouse dataset was obtained using micro-arrays, and also did not correlate with any of the other human datasets (**Figure 2**). There are two main factors that could explain this lack of correlation: the most likely is the technology platform used (dual microarray vs. RT-PCR: microarrays are less sensitive and quantitative than RT-qPCR and have a significantly lower dynamic range (18) although we cannot exclude a role for the geographic area of patients’ recruitment, India vs. Sub-Saharan Africa [there are different distributions of geographically restricted *Mtb* strains detected in the samples (28)]. The differences compared to the microarray dataset was also clear when the top 15% expressed *Mtb* genes of each published dataset were compared: only seven of the 90 *Mtb* top expressed genes which were shared in all RT-PCR datasets, were found to be highly expressed in the microarray dataset (**Figure 1B**). This discrepancy is evident also when comparing our findings to those obtained in a previous study (29) that analyzed the overlaps between upregulated genes in human and murine lungs identified from two microarray datasets (30, 31). In this meta-analysis only four genes were shared across species (Rv0316, Rv3347c, Rv3532, and Rv3706c), none of which is included in the top ranked transcripts of our analysis. Besides the fact that this data was based on microarray assays, the limited overlaps could be explained by the fact that in these studies the up-regulation of the genes is relative to the expression found in *in vitro* transcriptomics. Our findings differ also from another comparative analysis among *Mtb* transcriptomes from susceptible (I/StSnEgYCit) and resistant (C57BL/6YCit) mouse strains, which defined 209 commonly upregulated genes using the Audic-Claverie algorithm (32). However, from this list only 17 *Mtb* transcripts overlap with the top ranked genes found in our C3HeB/FeJ “Kramnik” dataset, a number that decreases to two if including in the comparison also the human datasets (**Data File S1**: **Table S1.10**). Therefore, we think it is crucial to use the same analysis pipeline when examining different datasets.

We also analyzed two additional *in vitro Mtb* transcriptome (RT-PCR based) datasets, both from log phase as well as from stationary phase cultured *Mtb* bacteria, and compared their gene expression profiles with those from the *in vivo* analyses. As expected, the *in vitro* log phase grown data correlated most strongly with the *in vivo* datasets (**Figure 2B**). However, despite the overall correlation, the *in vivo* pulmonary core *Mtb* transcript set only partially overlapped with the *in vitro* data, emphasizing the need for unbiased *in vivo* analyses to identify core *Mtb* transcriptome expression signatures that can inform the design of intervention tools, such as diagnostic tests, vaccines or new drugs. Of note, when taking only the top ranked transcripts from the *in vitro* datasets these had more overlap with the human datasets than those found between the two *in vitro* conditions. This finding may suggest that a combination of *in vitro* experimental models may translate better into the dynamic and heterogeneous state of *Mtb* during an *in vivo* infection (33).

The 90 *Mtb* top expressed genes were enriched for biological processes such as growth, translation, pathogenicity, protein secretion, and response to hypoxia and oxidative stress (**Figure 1C**). This trend was found also when genes were grouped according to previously described functional categories (11). In fact, the highest and most consistent expression ranks included virulence-associated *Mtb* genes, and were found among *esx* genes, ribosomal protein genes, genes related to metabolic pathways, and genes encoding proteins in response to hypoxic and oxidative stress, suggesting the presence of metabolic active bacilli in the samples investigated here (**Figure S1**). These signatures are compatible with active *Mtb* infection. Additionally, most of the 90 top expressed genes within the RT-PCR dataset were found expressed in independent and unbiased mouse and human derived RNA-Seq datasets. Unexpectedly, 35 out of these 90 highly abundant *Mtb* transcripts encode for proteins not yet been described as *Mtb* antigens or drug targets (**Figure 1B**) (7, 19). This key finding underscores how our integrative transcriptomic approach helps generate new hypotheses worth future investigation.

Although based on different mouse strains (C3H vs. BL6), and time of infection (five time points spanning from two to 12 weeks vs. two weeks only), the mouse lung RT-PCR dataset showed the highest correlation with the unbiased RNA-Seq datasets (**Figure 2A**) which detected, with only one exception (Rv3619c for the mouse derived dataset)¸ all genes encoding IVE-TB antigens. The latter had been selected based on high expression in the *Mtb* infected lungs of “Kramnik” mice, and the present analysis further extends this to human *Mtb* infected lung derived samples (**Figure 3A**) (8). By contrast, many of the *Mtb* genes encoding antigens identified only on the basis of their recognition in human peripheral blood, mostly showed lower expression than the IVE-TB antigens in the lung-centric datasets studied here (**Figure 3B**). Based on our hypothesis this suggests that only a subset of proteins immunogenic in human blood tests might have vaccine potential, but this needs to be demonstrated formally. However, one case in point is the failure of the MVA85 phase IIb trial which showed no additional efficacy of MVA85 booster vaccination on top of BCG in preventing TB in infants (34). We found that the encoded antigen, Ag85A (Rv3804c) used in MVA85A exhibited lower expression than for example antigens contained in the successful M72 vaccine (**Figure 3A**) (5), and this may underlie Rv3804c’s poor performance as single subunit vaccine antigen. Although the different protective efficacies of subunit vaccines are difficult to compare, antigen availability and presentation to immune cells in the respiratory tract of *Mtb* infected subjects may well be a key factor influencing their eventual efficacy. Of note, in our analysis, most promising TB subunit-vaccine candidates, including those in current clinical trials, contained at least one protein encoded by a highly expressed *Mtb* gene in multiple transcriptomic datasets.

We also performed further comparative analyses on genes encoding antigens that are currently used in commercial TB diagnostics or additional candidate vaccines (7). As expected, antigens implemented in approved TB diagnostics such as ESAT-6 and CFP-10 (Rv3875c and Rv3874) were highly or moderately expressed, except TB7.7 (Rv2654) that showed only low or undetectable expression levels. T cell reactivity towards TB7.7 has been investigated in short-term T cell cultures (35, 36) and in a direct *ex vivo* assay (37). The latter approach failed to find evidence for antigenicity of TB7.7 in a cohort of 18 LTBI donors (37). This result was unexpected given the presence of HLA-class II binding peptides in the antigen. Here we postulate that this lack of antigenicity in fact may reflect the low expression levels of TB7.7 which we find in the respiratory tract of untreated TB patients. Together, these findings support the use of other antigens for early detection of *Mtb* infection such as those recently proposed to be included in ESAT-6 free immunodiagnostic tests (Rv2348, Rv3615, and Rv3865) (23), which were instead found highly expressed (**Figure 3B**).

Another interesting application of our findings may be the refinement and implementation of new mega-pools of *Mtb* epitopes, a first version of which has already been tested in several cohorts of *Mtb* infected subjects (24, 25). Our analysis revealed that 19% (n=17) of the 89 *Mtb* genes encoding for those epitopes were poorly expressed *in vivo* (**Figure S1**). If confirmed also at a protein level, depleting new mega-pools of such peptides might help denoising the promiscuous immune reactivity found against the *Mtb* epitope mega-pool (24) and allow a better characterization of those peptides most likely available during infection.

A limitation of our analysis might be that differential gene expression does not by definition correlate with differential protein expression, and differential protein expression does not equal differential functionality. The analysis of large sets of genes as performed in this study, however, we think might have mitigated this risk, even though at the individual gene level exceptions may exist. Several *Mtb* proteomic datasets have been reported, confirming high expression of *Mtb* proteins known to be diagnostic or candidate vaccine antigens, but as far as we know this has not been correlated systematically to vaccine-, diagnostic- of drug-target efficacy. Furthermore, although *Mtb* transcript levels may not necessarily predict protein levels (38), they are helpful in understanding global transcriptome dynamics with impact on potential local antigen availability and presentation as well as drug target expression during *Mtb* infection. Such knowledge may help de-risking TB vaccine and drug development by selecting the most highly expressed and presented *Mtb* genes and proteins in the human lung during infection.

Currently there are 28 approved TB drugs which have been or are being prioritized and combined based on the sensitivity of *Mtb* to these drugs. Isoniazid, Rifampin (or rifapentine or Rifabutin), Ethambutol (administered for six months) and Pyrazinamide are used as first-line drugs for drug sensitive TB, while the rest are used in different regimens against multi- or extensively-drug resistant TB (MDR-TB and XDR-TB respectively) (http://bmi.icmr.org.in/tbdrugs/). An important aspect of our analysis is that it confirmed the high-medium expression of all 22 *Mtb* genes encoding targets of TB drugs efficacious in clinical settings (**Figures 4**, **S1**). These results indirectly validate our approach but, more importantly, support the value of using pulmonary *Mtb* transcriptomic datasets to select for new potential TB drug targets. Indeed, our work also reveals high expression of *Mtb* genes that have not been studied as drug target molecules, suggesting that the here reported approach could inform discovery of new drug targets.

Collectively, by our comparative analyses of *in vivo* pulmonary *Mtb* transcriptomes both from *Mtb* infected animals and humans significantly increase our understanding of *in situ* host-pathogen interactions in TB, and can help refining the *in vivo* expressed *Mtb* genes and proteins to accelerate and innovate TB vaccine, diagnostics and drug development. Enhanced focus on lung centric studies in TB should therefore be encouraged.

## Materials and Methods

### Study Design

The central aim of the study was to examine the gene expression profile of *Mtb* during *in vivo* pulmonary infection in humans and mouse models and compare those to the *Mtb in vitro* expression signature. We analyzed large scale transcriptomic datasets from *Mtb*-infected human sputa (n=35), human bronchoalveolar lavages (n=11), TB-hypersusceptible (C3H/FeJ) mouse lungs (n=20), alveolar macrophages from TB-resistant (C57BL/6J) mice (n=3) and compared data to *in vitro* cultured *Mtb* gene-expression profiles and to a RNA-Seq human sputum derived *Mtb* transcriptome dataset from TB patients (n=7) (**Table 1**). Within each dataset and each sample, a relative score was calculated by ranking the expression level of each gene relative to all genes assayed.

### Data Source and Data Process

#### Dataset a: RT-PCR *Mtb* Transcriptome Dataset From Lung of *Mtb* infected Mice

In brief, C3HeB/FeJ (C3H) mice, housed under specific pathogen-free conditions were infected with *Mtb* Erdman by aerosol challenge (25–50 CFU *Mtb* using a Madison chamber). Four mice per group were sacrificed at five different time points. Quantification of *Mtb* transcript profile was performed on 2068 genes, mostly representing the first gene of each predicted operon, as previously described (8). Total *Mtb* RNA was isolated from infected mouse lung tissue, after two, four, six, nine, and 12 weeks of *Mtb* infection. cDNA synthesis was conducted and cDNA further amplified *via* controlled multiplex pre-amplification. Sequences and design of PCR primer/probe sets are available at http://genes.stanford.edu/technology.php. Individual gene transcript quantification was carried out using TaqMan primer/probe sets (Biosearch Technology, Petaluma, CA, USA). For each time point after infection, the median cycle threshold values of four mice per strain were transformed to relative gene copy numbers (RGCNs) based on logarithmic transformation/linear regression equations devised from calibration curves (CT and RGCNs used in this paper are available in the **Data File S1**: **Table S1.1**) (8).

For each time point, a relative score was calculated by ranking the median RGCN of each gene to all genes assayed. A rank of 100 represents the most highly expressed gene, a rank of zero represents the lowest expressed gene. The same percentile rank values were assigned to genes with same median RGCN. Thereafter, the median relative expression score among all time points was calculated for each gene (**Data File S1**: **Table S1.1**).

#### Dataset b: RT-PCR *Mtb* Transcriptome Dataset From Human Sputum of Ugandan TB Patients

*Mtb* gene-based RT-PCR data were obtained from the supplementary files from the original paper (**Data File S1**: **Table S1.2**) (11). Briefly, in that study expectorated sputum of 17 Ugandan adults with untreated pulmonary TB was collected into a sterile cup containing guanidine thiocyanate solution for immediate RNA preservation. Total RNA was extracted and amplified. The authors quantified a total of 2406 *Mtb* transcripts *via* multiplex quantitative RT-PCR, and normalized mRNA expression data using a minimum-variance data-driven method. For each sample, we calculated a relative score by ranking the expression level of each gene to all genes assayed.

A rank of 100 represents the most highly expressed gene, a rank of zero the lowest expressed gene. For each gene, the median relative expression score was available for specimen collected before TB treatment and therefore used in this analysis (**Data File S1**: **Table S1.2**).

#### Dataset c1 and c2: RT-PCR *Mtb* Transcriptome Dataset From Human Sputum and BAL of South African TB Patients

*Mtb* gene-based RT-PCR data were obtained from supplementary files provided in the original paper (**Data File S1**: **Table S1.3**) (12). Briefly, in that study, human sputum (dataset c1) and BAL (dataset c2) samples were collected from 11 untreated South African TB patients. From these specimens, RNA extraction was performed using a phenol/chloroform protocol and transcriptional profiling of 1970 *Mtb* genes was assessed *via* multiplex quantitative RT-PCR (TaqMan) with a LightCycler 480 (Roche, Indianapolis, Indiana) (details in the original manuscript). Data were batch corrected using a median approach. Since BAL and sputa were paired samples, the *Mtb* transcriptional data were normalized using a previously-described minimum variance method (12) (**Data File S1**: **Table S1.3**).

For each time point and sample, a relative score was calculated by ranking the median CT level of each gene among all genes assayed. Rank of 100 represents the most highly expressed gene, a rank of zero the lowest expressed gene. Identical percentile rank values were assigned to genes with the same median CT (**Data File S1**: **Table S1.3**).

#### Dataset c3 and c4: RT-PCR *Mtb* Transcriptome Dataset From *In Vitro* Cultured *Mtb*

*Mtb* gene-based RT-PCR data were obtained from supplementary files provided in the original paper. Briefly, this study provided *Mtb* gene expression data from H37Rv cultured in log phase aerobic growth (four replicates) (dataset c3) and in non-replicating persistence state (NRP-2; 0·06% of oxygen in the culture) (six replicates) (dataset c4) (**Data File S1**: **Table S1.4**) (12). From these specimens, RNA extraction was performed using a phenol/chloroform protocol and transcriptional profiling of 2124 *Mtb* genes was assessed *via* multiplex quantitative RT-PCR (TaqMan) with a LightCycler 480 (Roche, Indianapolis, Indiana) (details in the original manuscript) (12).

For each time point and sample, a relative score was calculated by ranking the median CT level of each gene to all genes assayed. Rank of 100 represents the most highly expressed gene, a rank of zero the lowest expressed gene. Identical percentile rank values were assigned to genes with the same median CT (**Data File S1**: **Table S1.4**).

#### Dataset d: Microarray *Mtb* Transcriptome Dataset From Human Sputum From Untreated Indian TB Patients

Microarray data of 3924 *Mtb* genes were obtained from the files deposited in GEO database, accession number: GSE93316. Briefly, in this study human sputa from seven untreated TB patients were collected in Chandigarh, India and used for extraction of RNA by RNAZOL in Primestore. For DNA microarrays, the DNase treated RNA (50ng) was amplified using the MessageAmp™ II-Bacteria RNA Amplification Kit (Ambion^®^) and then reverse transcribed using superscript III RT. The amplified and labelled cDNA was then hybridized to *Mtb* arrays obtained from the Center for Applied Genomics (Public Health Research Institute; Newark, NJ). The microarrays were scanned with Axon 4000B scanner and processed further with GenePix Pro 6.1 software.

For our analysis, we used the within sample print-tip Lowess normalized fluorescence intensities (F635 medians from the original paper) detected from the cDNA of the smear positive samples. Quantile normalization was performed in R studio, as described in the methods below. Within each sample, genes were ranked from zero to 100, with zero and 100 representing respectively the lowest and the most highly expressed gene. The same percentile rank values were assigned to genes with same normalized fluorescence intensities. The median rank was calculated for each gene among the donors (n=seven) (**Data File S1**: **Table S1.5**).

#### Dataset e: RNA-Sequencing (RNA-Seq) *Mtb* Transcriptome Dataset From Alveolar Macrophages of *Mtb* Infected Mice

Normalized *Mtb* transcriptome data were obtained from the **Supplementary Figure 1** of the GEO depository (GSE132354). Briefly, as described in the depository, C57BL/6J mice (n=three) purchased from the Jackson Laboratory were infected with *Mtb* smyc’::mCherry Erdman for 14 days. The total mixed RNA enriched for bacterial reads was extracted following the protocol described in the paper (16). rRNA removal was performed using 50-100ng total RNA input and a modified protocol for the Ribo-Zero Epidemiology Gold rRNA removal kit (Illumina). Briefly, 90 μl bead stock was used per sample, together with two μl each of reaction buffer and removal solution in a 20 μl reaction volume, as detailed in the manufacturer’s protocol. The rRNA-depleted samples were purified by precipitating the RNA. Sequencing libraries were generated using the NEBNext Ultra™ II Directional RNA Library Prep Kit for Illumina (New England BioLabs). Libraries were sequenced on a NextSeq500 (Illumina) in multiple rounds until the desired sequencing depth for bacterial reads was reached (target 1M 85nt reads). Flexbar (v. 3.4) has been used to remove low quality reads and trim Illumina adapters. rRNA reads have been removed using Bowtie2 (sensitive mode). rRNA filtered fastq files were split using Bowtie2 (very-sensitive mode) into species-specific files using the two reference genomes, GRCm38.94 for *Mus musculus* and NCBI assembly GCA_00668235.1 for *Mtb Erdman* Hisat2 (v. 2.1.0). Raw read counts for each sample were obtained using HTSeq (v. 0.11.0).

A total of 3766 *Mtb* genes whose names could be assigned to the corresponding Rv numbers were considered in our analysis. Within each sample, genes were ranked from zero to 100, with zero and 100 representing respectively undetected and the most highly expressed gene. The same percentile rank values were assigned to genes with same raw read counts. The median of these ranks was calculated for each gene among samples (**Data File S1**: **Table S1.6**). Since this *in vivo* RNA-Seq dataset was derived only from an early stage of *Mtb* infection (two weeks), the data was used primarily to validate results obtained from the RT-PCR *Mtb* transcriptomes.

#### Dataset f: RNA-Sequencing (RNA-Seq) *Mtb* Transcriptome Dataset From Human Sputum of South African TB Patients

RNA-Seq libraries for the 7 sputum samples from 6 untreated South African TB-only and 1 TB-HIV patients were prepared with 200ng of corresponding RNA using the Ovation Human FFPE RNA-Seq Multiplex System (NuGen, San Carlos CA, USA). To guarantee immediate RNA preservation, the sputum was lysed in Trizol immediately after collection and total RNA was extracted using chloroform and purified and concentrated with the RNA Clean & Concentrator kit Zymo Research, Irvine CA, USA. Each sputum library was loaded onto a single lane in a flow cell and sequenced with a Hi-Seq 2500 instrument using SE100 reaction (Illumina, San Diego CA, USA).

The quality of the sequencing fastq files was analyzed using FastQC (v0.11.5). Sequence reads were adapter- and quality- trimmed using Trimmomatic (v0.36) before aligning to the human genome (Ensembl GRCh38 build 88) using STAR aligner (v2.5.2a). To assure that the reported RNA-Seq reads did not map to other commensal bacteria such as oral flora, the sequence reads were quality filtered and then aligned to the human genome, with unaligned reads extracted for microbiome taxonomy classification, species mapping and subsequently aligned to reference genomes of *Mtb* using STAR aligner (v2.5.2a). The alignment files were name sorted by SAMtools (v1.2) and gene expression was quantified using BEDTools (v.2.26.0). The RNA-Seq data reported in this paper have been deposited at GEO with the accession number GSE137518.

Quantile normalization was performed in R studio, as described further in these methods. Within each sample, genes were ranked from 0 to 100, with 0 and 100 representing respectively undetected and the most highly expressed gene. The same percentile rank values were assigned to genes with same raw read counts. The median of these ranks was calculated for each gene among donors (n=7) (**Data File S1**: **Table S1.7**). In the human sputum samples, although the number of detected *Mtb* genes varied between 2957 and 3993 (median=3716), many genes had both equal raw read counts and read counts below 100, thus skewing the expression rank towards the lower interquartile. Therefore, the *in vivo* RNA-Seq dataset was mostly used to validate results obtained from the RT-PCR *Mtb* transcriptomes.

### Quantile Normalization of RNA-Seq and Microarray Datasets

In order to compare microarray data among biological samples, read counts and signal intensities have to be adjusted to eliminate systematic effects that are not associated with the biological differences of interest. It has been shown that quantile-based normalization is a robust procedure to remove technical variations without introducing additional noise and to make distributions identical across samples (39). Quantile normalization was performed in R studio by applying the following function (40):

quantile_normalisation <- function(df)

{df_rank <- apply (df,2, rank, ties.method=“min”)

df_sorted <- data.frame (apply (df, 2, sort))

df_mean <- apply (df_sorted, 1, mean)

index_to_mean <- function (my_index, my_mean) {return (my_mean [my_index])}

df_final <- apply (df_rank, 2, index_to_mean, my_mean = df_mean)

rownames (df_final) <- rownames(df)

return(df_final)}

### GO and STRING Analysis

Gene ontology (GO) enrichment analysis was performed by using PANTHER Overrepresentation Test (21). The most expressed *Mtb* genes shared among the *Mtb* transcriptome datasets previously described were evaluated in respect to the *Mtb* genome (all genes in the database). P values were calculated by Fisher’s exact test adjusted for false discovery rate. Only biological processes significantly enriched (p value < 0·05) were reported. STRING (https://string-db.org/) was used to predict protein-protein interactions and show network connectivity (41). The network was based on evidence from experiments, curated databases or prediction of co-expression, and gene fusions (medium confidence score >= 0·4).

### Correlation Matrix

All datasets were compared in R using the ggcorrplot package (https://cran.r-project.org/web/packages/ggcorrplot/ggcorrplot.pdf). Spearmans rank correlation coefficient was computed based on the median rank of the 1813 *Mtb* genes investigated in all datasets. Since the datasets used in our analysis consist of a large sample size, all Spearmans rank correlation coefficients above 0.13 were significant (calculated using the cor.test function in R).

### Venn Diagrams

To show the numbers of top ranked *Mtb* genes that overlap between *in vivo* and *in vitro* datasets, Venn diagrams have been made using the ggVennDiagram and the ggplot2 packages (https://cran.r-project.org/web/packages/ggVennDiagram/index.html and https://cran.r-project.org/web/packages/ggplot2/index.html).

### Code Availability

Code is available in the Methods section and at the following link: (https://cran.r-project.org/web/packages/ggcorrplot/ggcorrplot.pdf).

## Data Availability Statement

The datasets presented in this study can be found in online repositories. The names of the repository/repositories and accession number(s) can be found in the article/**Supplementary Material**.

## Ethics Statement

The studies involving human participants were reviewed and approved by The Human Research Ethics Committee of the University of Cape Town (HREC References: 031/2012 and 568/2012). The patients/participants provided their written informed consent to participate in this study.

## Author Contributions

This project was developed and designed by MC and TO. MC performed the analyses. MC and TO interpreted the analyses and wrote the manuscript. RP-JL and RW shared the human derived RNA-Seq dataset, participated to the scientific discussion and reviewed the manuscript. All authors contributed to the article and approved the submitted version.

## Funding

EC HORIZON2020 TBVAC2020, Grant Agreement No. 643381(MC, TO); EC ITN FP7 VACTRAIN project (MC, TO); NWO-TOP project Grant Agreement No. 91214038 (TO). Francis Crick Institute funded by URI grant FC0010218, Cancer Research UK (FC0010218) and Wellcome (FC0010218) (RP-JL, RW); Wellcome grant 104803 (RW); Wellcome grant 203135 (RW).

## Author Disclaimer

The text represents the authors’ views and does not necessarily represent a position of the funders who will not be liable for the use made of such information.

## Conflict of Interest

The authors declare that the research was conducted in the absence of any commercial or financial relationships that could be construed as a potential conflict of interest.

The reviewer CLA declared a past co-authorship with one of the authors to the handling Editor.

## Publisher’s Note

All claims expressed in this article are solely those of the authors and do not necessarily represent those of their affiliated organizations, or those of the publisher, the editors and the reviewers. Any product that may be evaluated in this article, or claim that may be made by its manufacturer, is not guaranteed or endorsed by the publisher.

## Supplementary Material

Supplementary Figure 1 Expression level of *Mtb* genes belonging to specific categories previously defined in literature. Each heatmap lists *Mtb* genes belonging to previously defined functional categories (11, 22). Specific pathways are indicated on the right of the genes encoding proteins related to metabolic pathways. Each column represents the relative expression rank within each dataset. Datasets are listed from left to right with the following order: C3HeB/FeJ mouse lung dataset (8) **(A)**; human sputum (HS) dataset from Ugandan TB patients (10) **(B)**; HS **(C1)** and BAL **(C2)** from a cohort of South African TB patients (12); a microarray (MA) based dataset **(D)** from sputa of Indian TB patients (15). Red color coded Rvs indicate *Mtb* genes of the mega-pool which overlap with **Figure 3B**.

Supplementary Table 1 Source Data. This file contains nine supplementary tables for this paper. **Tables S1.1–S1.7** report the normalized data available for each of the published dataset. These datasets describe the expression of *Mtb* genes in: lung of infected C3HeB/FeJ (C3H)(S1.1) (8); alveolar macrophages (AM) of *Mtb* infected C57BL/6J mice (S1.6) (GEO: GSE132354) (16); human sputa (S1.2, S1.3, S1.5 and S1.7) (11, 12) (GSE137518); in BAL samples (S1.3) (12) from TB patients as well as under *in vitro* conditions (S1.4). If not performed previously, a quantile normalization was performed on raw data of interest (for data in S1.5). Within each dataset, genes were ranked from zero to 100, with zero and 100 representing respectively the lowest or undetected (only for the RNA-Seq data) genes and the most highly expressed gene. The median rank was calculated for each gene among samples. **Table S1.8** reports the top expressed *Mtb* genes in common among the human RT-PCR datasets only. **Table S1.9** reports the overlap between the top ranked *Mtb* genes of the two different experimental models with the top ranked *Mtb* genes of the *in vivo* RT-PCR datasets. **Table S1.10** lists the 17 *Mtb* transcripts overlapping with the top ranked genes found in our C3HeB/FeJ “Kramnik” dataset (8) and a previous published comparative analysis between *Mtb* transcriptomes from susceptible (I/StSnEgYCit) and resistant (C57BL/6YCit) mouse strains (32).
