# Supplementary material for: The In Vivo Transcriptomic Blueprint of Mycobacterium tuberculosis in the Lung
Source: Front Immunol. 2021 Dec 22;12:763364. doi: 10.3389/fimmu.2021.763364 (PMC8727759; doi:10.3389/fimmu.2021.763364)
Supplement: Supplementary Figure 1 — Expression level of Mtb genes belonging to specific categories previously defined in literature. Each heatmap lists Mtb genes belonging to previously defined functional categories (11, 22). Specific pathways are indicated on the right of the genes encoding proteins related to metabolic pathways. Each column represents the relative expression rank within each dataset. Datasets are listed from left to right with the following order: C3HeB/FeJ mouse lung dataset (8) (A); human sputum (HS) dataset from Ugandan TB patients (10) (B); HS (C1) and BAL (C2) from a cohort of South African TB patients (12); a microarray (MA) based dataset (D) from sputa of Indian TB patients (15). Red color coded Rvs indicate Mtb genes of the mega-pool which overlap with Figure 3B . [file DataSheet_1.pdf]

DosR regulon

	Mouse: Lung RT-PCR a	Human: Sputum RT-PCR b	Sputum RT-PCR c1	BAL RT-PCR c2	Sputum MA d
Rv0079		71	57	71	70
Rv0080	51	59	51	65	37
Rv0081	64	21	3	3	22
Rv0082	4	12	53	47	44
Rv0083	13	8			54
Rv0569	87	91	87	97	32
Rv0570	17	32	28	62	26
Rv0571c	64	69	52	67	65
Rv0572c	96	97	95	97	57
Rv0574c	63	42	6	27	52
Rv1733c	69	49	61	93	19
Rv1734c	97	66	41	49	46
Rv1736c	33	52	29	35	36
Rv1737c	57	52	60	59	27
Rv1738	100	99	95	99	89
Rv1812c	73	73	69	79	63
Rv1813c	79	36	15	27	1
Rv1996	85	15	2	5	20
Rv1997	53	33	24	51	19
Rv2003c	37	51	22	57	28
Rv2004c	82	81	54	61	43
Rv2005c	76	69	34	71	56
Rv2006	66	48	66	87	14
Rv2007c	99	91	63	81	29
Rv2028c	19	43	20	42	62
Rv2029c	82	66	76	95	48
Rv2030c	97	80	64	65	10
Rv2031c	100	92	97	99	22
Rv2032	80	87	90	98	49
Rv2623	94	82	56	88	13
Rv2624c	81	56	9	46	40
Rv2625c	95	27	9	33	78
Rv2626c	99	95	78	98	28
Rv2627c	98	59	4	10	23
Rv2628	55	48	41	83	44
Rv2629	94	60	31	36	77
Rv2630	90	71	32	45	24
Rv2631	7	34	34	89	24
Rv2830c	37	72	56	95	81
Rv3126c	51	39	1	19	17
Rv3127	95	82	70	88	21
Rv3128c	82	75	39	67	35
Rv3129	78	50	44	74	24
Rv3130c	98	87	82	97	42
Rv3131	96	63	68	82	28
Rv3132c	92	97	97	98	10
Rv3133c	85	75	65	74	60
Rv3134c	47	62	61	84	73

Rank: 0 20 40 60 80 100

Oxidative stress response

	Mouse: Lung RT-PCR a	Human: Sputum RT-PCR b	Sputum RT-PCR c1	BAL RT-PCR c2	Sputum MA d
Rv0324	1	21	25	33	60
Rv0338c	80	64	73	63	71
Rv0816c		60			53
Rv0823c	93	93	97	98	6
Rv0848	23	38	6	28	33
Rv1049	55	57	64	44	32
Rv1129c	80	91	94	84	24
Rv1285	63	40	23	27	80
Rv1286	90	81	35	68	91
Rv1317c	19	42	88	78	66
Rv1463		86			46
Rv1471	88	84	72	73	48
Rv1674c		6	13	13	71
Rv1908c	87	77	77	91	32
Rv1909c	99	89	61	72	31
Rv2374c	69	14	12	10	57
Rv2377c		52			20
Rv2380c	98	90	54	55	24
Rv2381c	96	62	20	58	21
Rv2382c	99	80	66	37	33
Rv2384	50	42	55	89	1
Rv2386c	82	36	8	1	3
Rv2621c	91	79	91	95	58
Rv2710	99	82	39	35	10
Rv2737c		4			9
Rv2745c	77	91	97	98	56
Rv2989	4	10	6	7	12
Rv3202c	10	13			28
Rv3223c	87	28	28	34	15
Rv3334	84	61	26	31	48
Rv3583c	98	99	100	99	30
Rv3585	26	16	5	2	19
Rv3841	95	98	99	99	12
Rv3913	70	85	73	79	23
Rv3914	99	100	99	100	82

Rank: 0 20 40 60 80 100

Enduring hypoxic response

	Mouse: Lung RT-PCR a	Human: Sputum RT-PCR b	Sputum RT-PCR c1	BAL RT-PCR c2	Sputum MA d
Rv0048c	46	71	75	58	65
Rv0084	7	33	19	34	37
Rv0116c	87	82	58	33	70
Rv0140	97	93	87	91	30
Rv0171	57	80	83	83	20
Rv0186	53	13	12	9	30
Rv0188	98	80	33	66	38
Rv0196	21	19	49	40	67
Rv0232	39	55	85	88	77
Rv0233	61	54	53	40	33
Rv0244c	99	72	84	80	53
Rv0251c	90	97	96	98	19
Rv0268c	70	33	14	6	69
Rv0324	1	21	25	33	60
Rv0327c	64	54	12	24	67
Rv0328	42	64	64	75	69
Rv0347	71	68	64	31	37
Rv0384c	5	27	33	61	32
Rv0474	88	70	95	85	14
Rv0494	3	47	78	93	46
Rv0520	59	60	27	11	31
Rv0521c	29	56	42	43	19
Rv0551c	53	52	23	29	33
Rv0563	84	84	65	91	13
Rv0575c	36	29	49	59	67
Rv0614	7	36	66	23	30
Rv0653c	34	53	57	50	64
Rv0654	48	9	5	6	87
Rv0687	61	55	34	29	49
Rv0726c	56	19	24	14	43
Rv0753c	27	43	21	27	20
Rv0754	10	20	4	13	76
Rv0757	87	73	57	47	10
Rv0766c	33	56	32	46	5
Rv0767c	21	49	55	90	77
Rv0792c	59	21	16	13	47
Rv0793	40	23	27	51	47
Rv0826	91	73	33	30	37
Rv0827c	31	35	50	46	8
Rv0847	24	24	10	19	34
Rv0967	92	60	51	64	11
Rv0986	95	73	55	50	98
Rv0989c	83	28	10	11	21
Rv0991c	100	93	91	96	29
Rv1048c	45	37	35	29	51
Rv1082	66	72	61	39	55
Rv1088	9	21			19
Rv1152	76	79	64	79	14
Rv1169c	82	36	4	4	42
Rv1221	100	96	94	85	43
Rv1243c	2	6			56
Rv1255c	13	30	15	18	10
Rv1256c	40	56	69	80	35
Rv1284	99	98	96	98	33
Rv1375	78	48	62	38	30
Rv1403c	93	73	58	76	28
Rv1405c	76	88	88	85	47
Rv1471	88	84	72	73	48
Rv1472	38	69	58	63	15
Rv1578c		4			48
Rv1623c	92	12	8	8	29
Rv1628c	14	27	53	44	49
Rv1652	40	36	19	42	50
Rv1669	43	15			51
Rv1733c	69	49	61	93	19
Rv1755c	47	37			28
Rv1760	37	21			47
Rv1766	58	66	63	60	55
Rv1806	95	14	1	1	23
Rv1874	37	30	4	13	70
Rv1875	43	16	5	4	24
Rv1888c	89	95	93	96	83
Rv1891	42	17	27	33	43
Rv1909c	99	89	61	72	31
Rv1926c	86	73	59	65	75
Rv1954c	65	66	28	55	17
Rv1955	92	73	31	47	6
Rv1956	88	64	17	36	35
Rv1960c	63	47	36	14	9
Rv1963c	18	27	4	30	1
Rv1974	45	36	7	33	49
Rv1985c	46	75	66	60	25
Rv1990c	90	97	96	94	51
Rv1994c	83	80	68	65	61
Rv2012	60	30	20	16	16
Rv2022c	45	23	10	8	11
Rv2034		3			39
Rv2050	77	92	84	93	17
Rv2122c		3			15
Rv2123	77	16	1	2	36
Rv2185c	87	90	95	92	25
Rv2243	88	18	1	9	93
Rv2299c	80	88	92	64	22
Rv2324		2			82
Rv2359	77	65	42	30	35
Rv2374c	69	14	12	10	57
Rv2389c	93	40	9	8	61
Rv2390c	87	41	59	55	30
Rv2412	70	93	99	98	75
Rv2465c		2			7
Rv2466c	84	86	81	94	2
Rv2497c		30	12	32	43
Rv2498c	13	15	13	2	54
Rv2499c	66	48	61	38	94
Rv2500c	88	63	20	23	44
Rv2504c	57	65	43	7	7
Rv2517c	93	58	17	12	40
Rv2526	51	4	21	67	67
Rv2557	94	36	8	14	70
Rv2558	36	22	28	79	70
Rv2583c		58	75	81	15
Rv2617c	97	97	88	95	12
Rv2626c	99	95	78	98	28
Rv2627c	98	59	4	10	23
Rv2628	55	48	41	83	44
Rv2642	60	58	50	89	61
Rv2653c	34	30	14	74	28
Rv2657c	95	78	36	28	80
Rv2660c	93	92	96	92	12
Rv2662	60	46	20	42	62
Rv2663	95	89	63	61	38
Rv2687c	25	69	29	7	71
Rv2688c	24	41	13	54	28
Rv2693c	35	22	10	24	81
Rv2694c	81	84	77	73	16
Rv2699c	96	94	85	91	5
Rv2700	24	32	52	40	22
Rv2744c	87	93	96	90	18
Rv2745c	77	91	97	98	56
Rv2830c	37	72	56	95	81
Rv2876	92	84	92	93	61
Rv2913c	62	51	38	77	8
Rv3007c	52	72	77	40	75
Rv3016	89	31	8	3	18
Rv3066	31	41	33	25	54
Rv3161c	76	11	37	6	19
Rv3176c	10	10			13
Rv3188	50	79	76	88	7
Rv3197A	28	42	75	86	
Rv3206c	88	80	63	75	31
Rv3221A	13	40	75	68	55
Rv3223c	87	28	28	34	15
Rv3290c	57	48	32	56	46
Rv3334	84	61	26	31	48
Rv3406	49	49	90	87	28
Rv3418c	94	100	100	100	41
Rv3480c	28	39	58	37	4
Rv3503c	87	97	97	96	37
Rv3515c	92	80	11	8	8
Rv3531c	67	87	84	84	11
Rv3537	54	80	80	89	6
Rv3571	20	38	81	78	13
Rv3574	82	74	67	81	42
Rv3681c	91	67	40	39	64
Rv3747	89	48	39	28	2
Rv3848	1	19			9
Rv3862c	86	87	59	79	22
Rv3864	99	65	33	33	9
Rv3913	70	85	73	79	23

Rank: 0 20 40 60 80 100

Phage proteins and insertion sequences

	Lung RT-PCR a	Sputum RT-PCR b	Sputum RT-PCR c1	BAL RT-PCR c2	Sputum MA d
Rv0094c		88			78
Rv0095c	21	61	21	54	75
Rv0336	66	87	86	76	9
Rv0397	2	6	34	9	99
Rv0605	57	62	52	55	31
Rv0741	49	18	28	17	38
Rv0829	36	19	21	20	39
Rv0920c	19	8	13	17	4
Rv1034c		3			64
Rv1036c	23	36	92	80	65
Rv1047	23	79	93	75	61
Rv1054	16	52	45	19	40
Rv1128c	37	82	92	93	19
Rv1148c	76	66	65	59	31
Rv1150	30	83	77	67	44
Rv1313c	19	73	70	74	63
Rv1369c	8	41	23	22	27
Rv1573		5			43
Rv1577c		79			11
Rv1578c		4			48
Rv1581c		63			59
Rv1584c	38	65	0	3	31
Rv1588c	48	81	89	76	72
Rv1701	21	27	79	88	34
Rv1702c	74	33	19	25	78
Rv2013		47			53
Rv2085	12	23			28
Rv2087	14	11	12	14	15
Rv2309c	10	22			25
Rv2355	9	49	20	16	29
Rv2647	84	33	23	60	34
Rv2650c	5	15	7	1	27
Rv2651c	57	63	77	75	7
Rv2652c	2	29	68	74	9
Rv2653c	34	30	14	74	28
Rv2655c	32	46	29	4	13
Rv2656c	7	37	33	55	38
Rv2657c	95	78	36	28	80
Rv2659c	82	62	11	60	83
Rv2666		96			43
Rv2792c	21	47	41	25	17
Rv2810c	5	16			60
Rv2943	9	21			58
Rv2961	17	18	23	19	32
Rv2979c		2			52
Rv3349c	2	16			89
Rv3386	20	19	11	24	60
Rv3427c		4			93
Rv3428c	9	8			31
Rv3431c	25	63	43	55	22
Rv3640c	13	7			60
Rv3750c	76	48	35	34	45
Rv3751	25	30			30
Rv1765A	67	51	58	37	
Rv3770B	17	18	23	24	

Rank: 0 20 40 60 80 100

TB drug targets

	Lung RT-PCR a	Sputum RT-PCR b	Sputum RT-PCR c1	BAL RT-PCR c2	Sputum MA d
Rv3601c	96	97	89	96	86
Rv1305	96	95	98	96	62
Rv0005	85	94	96	97	2
Rv2043c	87	94	93	94	13
Rv2523c					90
Rv3854c	95	87	96	88	27
Rv1630	92	87			17
Rv3423c					84
Rv1484	78	87	75	80	36
Rv1908c	87	77	77	91	32
Rv0206c	91	77	53	40	81
Rv0667	72	74	75	63	44
Rv3806c					69
Rv3794	71	62	32	36	64
Rv3795					61
Rv1979c	43	64	60	8	90
Rv2447	20	88	57	41	56
Rv2196		99			13
Rv2163c	12	54	69	58	15
Rv2416c	54	60	34	34	99
Rv3262	14	52	54	66	38
Rv0407	69	52	49	45	87
Rv3790					51
Rv0006	83	64	50	33	26
Rv3608	8	47	52	77	10
Rv3547	81	72	38	46	46
Rv2156c	42	61	44	26	81
Rv2068c					42
Rv1173	31	41	77	69	20
Rv0706	40	52	31	33	38
Rv0701					37
Rv2535c					37
Rv0678	53	10	32	17	54
Rv3793					31
Rv2981c	15	35	20	57	27
Rv2524c	27	29	20	23	58
Rv0503c	78	26	25	13	31
Rv3261	28	18	26	20	54
Rv0682					25
Rv2763c	25	24			30
Rv1302	63	19	3	3	36
Rv0050	42	30	11	7	7
Rv1267c	30	6	4	8	42
Rv0132c	46	7	3	5	45

Rank: 0 20 40 60 80 100

Toxins and antitoxins

	Lung RT-PCR a	Sputum RT-PCR b	Sputum RT-PCR c1	BAL RT-PCR c2	Sputum MA d
Rv0239		99			62
Rv0240		72			58
Rv0298		97			10
Rv0299		86			84
Rv0300	81	71	81	78	59
Rv0301		78			54
Rv0549c		83			68
Rv0550c	5	10			29
Rv0581		15			13
Rv0582		88			38
Rv0595c	46	51	68	47	25
Rv0596c	51	64	75	73	50
Rv0598c		79			61
Rv0599c	80	36	40	27	70
Rv0608	81	69	76	37	11
Rv0609		81			18
Rv0617		85			79
Rv0623		76			13
Rv0624		88			90
Rv0626		71			28
Rv0627		65			58
Rv0656c	16	52	35	22	54
Rv0657c	55	68	61	59	29
Rv0659c		97			30
Rv0660c		90			58
Rv0661c		12			35
Rv0662c		14			88
Rv0664		12			72
Rv0665		59			34
Rv0748		61			26
Rv0749		88			34
Rv0918		4	0	0	5
Rv0919		98			83
Rv1102c		83			28
Rv1103c	33	62	80	84	20
Rv1114		89			9
Rv1241		84			18
Rv1242		86			63
Rv1246c		88			42
Rv1247c	82	89	92	86	15
Rv1397c		86			39
Rv1398c	99	100	99	99	82
Rv1494		90			13
Rv1495		71			17
Rv1560		88			18
Rv1561		87			44
Rv1720c		51			27
Rv1721c	31	49	47	75	41
Rv1740		85			40
Rv1741		62			23
Rv1838c		56			12
Rv1839c		51			58
Rv1942c		91			53
Rv1943c		74			32
Rv1952		93			23
Rv1953		85			3
Rv1955	92	73	31	47	6
Rv1956	88	64	17	36	35
Rv1959c		74			30
Rv1960c	63	47	36	14	9
Rv1989c		90			39
Rv1990c	90	97	96	94	51
Rv2009	80	74	76	41	21
Rv2010		94			60
Rv2018	21	46	44	40	10
Rv2021c		92			16
Rv2022c	45	23	10	8	11
Rv2034		3			39
Rv2035		62			19
Rv2103c		37			10
Rv2104c	42	75	78	58	34
Rv2493		76			64
Rv2494		80			81
Rv2526	51	4	21	67	67
Rv2527		90			12
Rv2530c		86			39
Rv2546	25	31	27	46	42
Rv2547		91			29
Rv2548		16			11
Rv2549c	61	85	67	60	44
Rv2550c		31			37
Rv2595	80	74	74	64	6
Rv2596		85			46
Rv2602		93			17
Rv2653c	34	30	14	74	28
Rv2757c		81			7
Rv2758c		61			13
Rv2759c		11			17
Rv2760c		9	21	12	10
Rv2829c		39			20
Rv2830c	37	72	56	95	81
Rv2865		89			6
Rv2866		70			16
Rv2871	48	8	5	4	16
Rv2872		48			16
Rv3180c		64			14
Rv3181c	54	14	1	4	32
Rv3183		48			40
Rv3188	50	79	76	88	7
Rv3189		73			16
Rv3320c		97			71
Rv3321c	66	73	91	79	36
Rv3357	52	36	48	64	36
Rv3358		64			24
Rv3384c		62			53
Rv3385c		8			15
Rv3407	97	77	67	83	81
Rv3408		88			32
Rv3749c		92			13
Rv3750c	76	48	35	34	45
Rv2601A	68	31	24	28	
Rv2530A		93			

Rank: 0 20 40 60 80 100

Metabolic related pathways

		Lung RT-PCR a	Sputum RT-PCR b	Sputum RT-PCR c1	BAL RT-PCR c2	Sputum MA d
Aerobic respiration	Rv1542c	33	30	20	11	39
	Rv1854c	95	86	71	70	15
	Rv1872c	94	95	95	78	80
	Rv2193	94	78	65	47	25
	Rv2194	93	72	55	59	92
	Rv2195		99			18
	Rv2196		99			13
	Rv2249c		71			73
	Rv2470	82	87	88	91	62
	Rv3145	27	69	79	81	38
	Rv3146	71	70	44	69	15
	Rv3147	60	54	48	45	30
	Rv3148		2	53	34	16
	Rv3149	43	18	12	8	24
	Rv3150	1	11			21
	Rv3151	44	33	42	49	24
	Rv3152	55	65	57	42	3
	Rv3153	20	69	65	72	11
	Rv3154	45	76	71	37	23
	Rv3155	75	17	10	13	9
	Rv3156	60	55	28	69	61
	Rv3157	58	69	36	29	17
	Rv3158	77	54	51	52	13
TCA cycle	Rv0066c	93	93	75	77	18
	Rv0889c	53	67	64	67	29
	Rv0896		99			33
	Rv0951	90	40	14	26	40
	Rv0952	87	79	80	70	36
	Rv1098c	61	7	1	0	74
	Rv1131	98	100	99	99	22
	Rv1240		88			79
	Rv1248c	86	71	52	52	36
	Rv1475c	68	74	86	82	59
	Rv2215	91	96	91	95	32
	Rv2498c	13	15	13	2	54
	Rv2967c		85			24
	Rv3316	90	78	24	53	72
	Rv3318	47	37	37	19	15
	Rv3339c	76	58	16	25	12
ATP synthesis	Rv1304	97	58	48	25	3
	Rv1305	96	95	98	96	62
	Rv1308	95	28	18	8	17
Anaerobic respiration	Rv0252	70	16	6	21	83
	Rv1161	88	90	96	97	52
	Rv1163	17	51	35	48	41
	Rv1552	41	24	9	40	30
	Rv1736c	33	52	29	35	36
Glyoxylate bypass	Rv0467	97	100	99	100	26
	Rv1837c	88	71	67	56	84
	Rv1915	96	99	98	99	95
	Rv1916		87			10
Triacylglycerol synthases	Rv3088		53			47
	Rv3130c	98	87	82	97	42
	Rv3234c	56	66	67	55	32
	Rv3734c	80	56	66	68	39
Polyketide synthases	Rv1013	93	86	65	82	50
	Rv1180	85	38	38	35	17
	Rv1181	85	84	39	48	11
	Rv1182	96	95	89	87	11
	Rv1527c	27	36	54	74	15
	Rv1528c	58	23	1	5	8
	Rv1660	79	88	74	65	12
	Rv1661	56	44	61	65	65
	Rv1662	7	20			18
	Rv1663	62	67	18	52	39
	Rv1664	47	82	55	57	28
	Rv1665	6	49	32	46	2
	Rv2377c		52			20
	Rv2380c	98	90	54	55	24
	Rv2381c	96	62	20	58	21
	Rv2382c	99	80	66	37	33
	Rv2384	50	42	55	89	1
	Rv2928	90	75	30	27	7
	Rv2931	96	96	82	87	26
	Rv2932	93	85	46	66	14
	Rv2934	95	81	77	66	29
	Rv2935	87	83	57	72	26
	Rv2939	92	95	70	67	22
	Rv2940c	82	78	97	69	35
	Rv2946c	28	64	75	49	86
	Rv2947c	69	60	95	64	43
	Rv3800c	15	74	80	72	33
	Rv3820c	78	40	11	13	78
	Rv3824c	90	66	20	12	79
	Rv3825c	71	83	74	61	46

Rank: 0 20 40 60 80 100

Transcription

	Mouse Lung RT-PCR a	Human Sputum RT-PCR b	Human Sputum RT-PCR c1	Human BAL RT-PCR c2	Human Sputum MA d
Rv0014c	86	49	40	24	66
Rv0015c	58	61	31	49	91
Rv0018c	63	78	50	48	13
Rv0019c	57	68	38	16	40
Rv0020c	78	83	79	83	33
Rv0022c		25			63
Rv0042c	29	72	43	47	49
Rv0043c		25			50
Rv0078	8	28	53	50	81
Rv0081	64	21	3	3	22
Rv0117	71	42	15	21	89
Rv0135c	66	81	89	71	82
Rv0144	10	19	18	15	92
Rv0158	50	67	62	37	11
Rv0165c	54	73	47	51	87
Rv0195		93			54
Rv0196	21	19	49	40	67
Rv0212c	50	43	18	5	49
Rv0232	39	55	85	88	77
Rv0238	52	63	39	40	56
Rv0260c	19	23			48
Rv0273c	79	72	88	85	10
Rv0275c	41	55	50	33	16
Rv0302	38	40	35	17	29
Rv0324	1	21	25	33	60
Rv0328	42	64	64	75	69
Rv0339c	24	34	75	65	34
Rv0386	40	16	18	16	73
Rv0410c	78	77	64	84	17
Rv0452	10	19	28	32	33
Rv0465c	43	52	95	97	52
Rv0474	88	70	95	85	14
Rv0490		93			34
Rv0491	86	81	72	78	55
Rv0494	3	47	78	93	46
Rv0576	35	25	10	11	11
Rv0586	27	85	56	29	44
Rv0600c		46			30
Rv0601c		4			83
Rv0602c	29	9			48
Rv0653c	34	53	57	50	64
Rv0681	23	26	85	69	42
Rv0691c	39	15			28
Rv0737		2			23
Rv0757	87	73	57	47	10
Rv0758	44	39	24	19	68
Rv0792c	59	21	16	13	47
Rv0818	16	5	1	2	20
Rv0823c	93	93	97	98	6
Rv0827c	31	35	50	46	6
Rv0844c	23	74	60	70	42
Rv0845	27	42	39	65	29
Rv0880	2	40	43	52	46
Rv0894	28	60	61	17	6
Rv0902c		35			43
Rv0903c	80	14	9	6	71
Rv0967	92	60	51	64	11
Rv0981		88			56
Rv0982		49			13
Rv1019	37	55	47	44	38
Rv1028c	47	46	50	30	42
Rv1032c		4			53
Rv1033c		86			37
Rv1049	55	57	64	44	32
Rv1129c	80	91	94	84	24
Rv1151c	36	35	33	35	51
Rv1152	76	79	64	79	14
Rv1167c	66	54	35	18	36
Rv1219c	28	45	71	66	13
Rv1255c	13	30	15	18	10
Rv1266c	78	65	69	83	42
Rv1267c	30	6	4	8	42
Rv1358	22	6			20
Rv1359	24	16	18	28	83
Rv1395	50	32	10	8	53
Rv1404	91	84	87	75	40
Rv1423	27	55	72	43	43
Rv1453	21	42			49
Rv1473A	42	71	66	60	
Rv1474c	53	60	76	71	7

	a	b	c1	c2	d
Rv1479	95	86	69	72	68
Rv1556	55	62	43	50	40
Rv1626	81	53	63	37	69
Rv1674c		6	13	13	71
Rv1675c	16	38	52	31	71
Rv1743	73	66	60	77	49
Rv1776c	15	34	80	90	61
Rv1816	27	40	33	21	32
Rv1846c	97	85	47	52	94
Rv1909c	99	89	61	72	31
Rv1931c	13	11	9	51	17
Rv1963c	18	27	4	30	1
Rv1985c	46	75	66	60	25
Rv1990c	90	97	96	94	51
Rv1994c	83	80	68	65	61
Rv2011c		74			38
Rv2021c		92			16
Rv2027c	51	40	62	56	3
Rv2034		3			39
Rv2088	51	48	55	13	78
Rv2160c	45	4	11	20	29
Rv2175c	10	17	19	10	67
Rv2232	16	36	41	44	55
Rv2250c	8	35	11	18	8
Rv2258c	41	81	82	70	39
Rv2282c	20	7	3	6	20
Rv2324		2			82
Rv2359	77	65	42	30	35
Rv2488c		19			36
Rv2506	50	80	97	73	24
Rv2621c	91	79	91	95	58
Rv2642	60	58	50	89	61
Rv2711	82	67	19	65	99
Rv2718c	74	66	55	58	3
Rv2720		89			1
Rv2745c	77	91	97	98	56
Rv2779c	44	30			8
Rv2884	27	41	39	58	34
Rv2887	35	25	22	17	24
Rv2914c		3			52
Rv2989	4	10	6	7	12
Rv3050c	81	88	81	86	29
Rv3058c	61	66	62	60	90
Rv3060c	47	40	40	34	17
Rv3066	31	41	33	25	54
Rv3095	64	68	93	85	45
Rv3124	34	34	85	73	17
Rv3132c	92	97	97	98	10
Rv3133c	85	75	65	74	60
Rv3143	50	50	60	34	37
Rv3160c	88	60	89	58	73
Rv3167c		6	12	10	20
Rv3183		48			40
Rv3197A	28	42	75	86	
Rv3208	67	34	42	29	57
Rv3219	99	99	98	96	31
Rv3220c	53	78	69	83	17
Rv3245c	76	86	74	83	72
Rv3246c	95	84	64	60	53
Rv3260c	77	82	82	80	24
Rv3291c	82	84	61	60	8
Rv3295	45	52	57	62	36
Rv3334	84	61	26	31	48
Rv3405c	6	43	26	6	41
Rv3416	93	73	90	75	83
Rv3557c	35	98	98	88	4
Rv3574	82	74	67	81	42
Rv3575c	14	24	21	36	46
Rv3583c	98	99	100	99	30
Rv3676	81	26	31	14	58
Rv3681c	91	67	40	39	64
Rv3736	27	45	54	45	20
Rv3744	67	82	84	63	22
Rv3764c	6	25	70	93	22
Rv3765c	57	80	76	76	26
Rv3830c	13	18	65	53	24
Rv3833	44	40	39	17	37
Rv3849	88	45	25	15	12
Rv3855	74	66	36	48	42
Rv3862c	86	87	59	79	22

Rank 0 20 40 60 80 100

Translation and sigma factors

	Mouse Lung RT-PCR a	Human Sputum RT-PCR b	Human Sputum RT-PCR c1	Human BAL RT-PCR c2	Human Sputum MA d
Rv0053	88	34	54	47	25
Rv0055		80			10
Rv0105c		33			46
Rv0182c	67	52	70	61	14
Rv0445c	57	35	6	5	70
Rv0640	97	90	92	96	51
Rv0641	77	89	95	87	12
Rv0651	36	47	62	44	23
Rv0700	88	92	91	87	15
Rv0704	90	95	90	95	71
Rv0705	88	71	58	28	40
Rv0706	40	52	31	33	38
Rv0707	49	45	45	32	86
Rv0708	88	76	73	73	54
Rv0709	92	94	95	95	70
Rv0710	86	90	96	86	68
Rv0714	75	70	59	37	73
Rv0715	94	97	98	97	91
Rv0716	83	85	94	71	30
Rv0717	89	14	4	3	44
Rv0718	82	90	83	85	70
Rv0735	32	48	9	26	33
Rv0995	11	49	58	80	41
Rv1015c	29	45	63	55	21
Rv1189	10	25	5	50	37
Rv1221	100	96	94	85	43
Rv1298	97	98	99	96	21
Rv1630	92	87			17
Rv1642	58	71	86	64	30
Rv2055c		79			3
Rv2056c		84			94
Rv2057c		93			21
Rv2058c		90			55
Rv2069	59	12	7	2	70
Rv2412	70	93	99	98	75
Rv2442c	64	53	79	56	53
Rv2703	84	69	46	39	25
Rv2710	99	82	39	35	10
Rv2890c	89	95	96	97	44
Rv2904c		6			84
Rv2909c	80	52	31	24	57
Rv3223c	87	28	28	34	15
Rv3241c	54	79	88	90	55
Rv3286c	9	67	58	79	73
Rv3328c	46	83	44	70	13
Rv3414c	9	23	16	9	20
Rv3420c		2			85
Rv3443c	82	70	65	77	23
Rv3458c	83	79	83	88	17
Rv3459c	58	50	59	16	27
Rv3460c	92	86	98	92	60
Rv3911		3			44

Rank 0 20 40 60 80 100

Esx genes

	Mouse Lung RT-PCR a	Human Sputum RT-PCR b	Human Sputum RT-PCR c1	Human BAL RT-PCR c2	Human Sputum MA d
Rv0287	99	97	99	99	85
Rv0288	99	98	94	98	71
Rv1037c	97	91	94	93	21
Rv1038c	99	99	100	100	54
Rv1197	98	100	100	100	74
Rv1198	43	42	17	11	70
Rv1792	99	100	100	100	62
Rv1793	97	92	95	95	64
Rv2346c	97	92	94	93	57
Rv2347c	98	100	100	100	80
Rv3017c	43	41	26	49	30
Rv3019c		8			77
Rv3020c	1	56	5	7	46
Rv3444c	1	13	5	8	21
Rv3445c	10	21	12	69	25
Rv3619c	97	92	93	95	59
Rv3620c	98	99	100	100	74
Rv3874	95	100	100	100	28
Rv3875	100	95	91	86	18
Rv3890c	95	60	70	84	50
Rv3891c	83	64	56	73	89
Rv3904c		5	7	32	41
Rv3905c	1	6			68

Rank 0 20 40 60 80 100

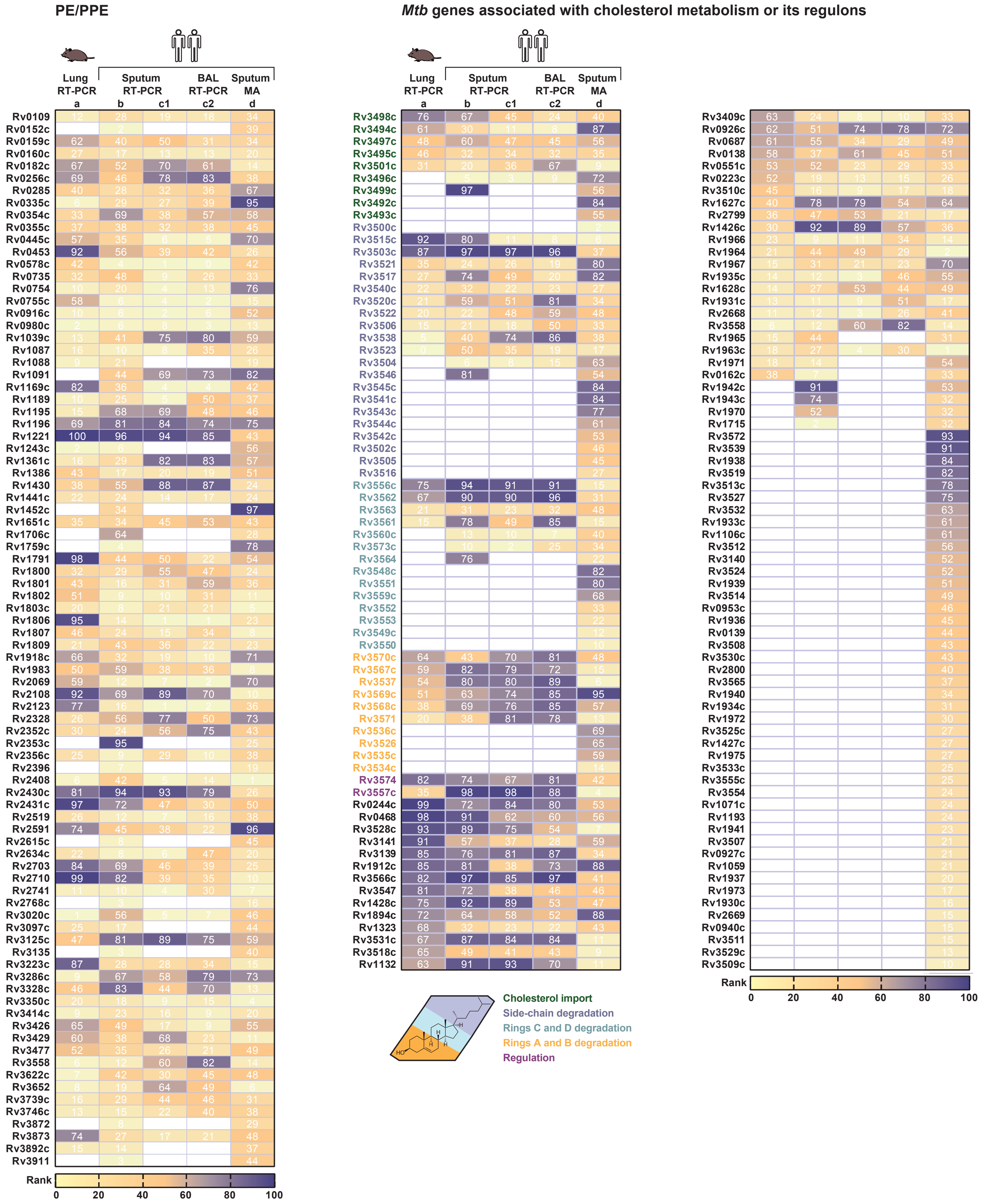
PE/PPE
Mtb genes associated with cholesterol metabolism or its regulons
Lung RT-PCR a
Sputum RT-PCR b c1
BAL RT-PCR c2
Sputum MA d
Rank
0 20 40 60 80 100
Cholesterol import
Side-chain degradation
Rings C and D degradation
Rings A and B degradation
Regulation

MegaPool

	Lung RT-PCR a	Sputum RT-PCR b	Sputum RT-PCR c1	BAL RT-PCR c2	Sputum MA d
Rv3418c	94	100	100	100	41
Rv3874	95	100	100	100	28
Rv3620c	98	99	100	100	74
Rv3615c	100	99	99	99	19
Rv3023c					99
Rv0287	99	97	99	99	85
Rv0288	99	98	94	98	71
Rv2031c	100	92	97	99	22
Rv1199c					97
Rv0985c	84	97	99	96	93
Rv3115					96
Rv1793	97	92	95	95	64
Rv1037c	97	91	94	93	21
Rv2346c	97	92	94	93	57
Rv3619c	97	92	93	95	59
Rv3022c					92
Rv2660c	93	92	96	92	12
Rv3875	100	95	91	86	18
Rv2512c					89
Rv0299		86			84
Rv0294					84
Rv3018c					83
Rv0129c	85	80	78	87	15
Rv2873	97	80	66	85	17
Rv2996c					79
Rv1908c	87	77	77	91	32
Rv1886c	77	80	76	81	31
Rv1047	23	79	93	75	61
Rv1196	69	81	84	74	75
Rv3125c	47	81	89	75	59
Rv2875	96	73	53	77	35
Rv1926c	86	73	59	65	75
Rv0442c					72
Rv2666		96			43
Rv0256c	69	46	78	83	38
Rv2874	67	45	87	94	57
Rv0290	70	76	66	63	39
Rv1317c	19	42	88	78	66
Rv3804c	82	69	59	60	49
Rv1172c					58
Rv3621c					58
Rv1361c	16	29	82	83	57
Rv0297					55
Rv0280					54
Rv0125	54	60	54	64	51
Rv0298		97			10
Rv0987		95			8
Rv0291	27	39	51	73	75
Rv3478					50
Rv1791	98	44	50	22	54
Rv3012c	18	51	49	38	67
Rv1788					49
Rv1195	15	68	69	48	46
Rv3330	6	41	47	94	86
Rv1706c		64			28
Rv3019c		8			77
Rv0289					42
Rv1198	43	42	17	11	70
Rv0453	92	56	39	42	26
Rv1366	41	39			16
Rv3025c					38
Rv2853					35
Rv0292					34
Rv3876	81	33	18	24	49
Rv1800	32	29	55	47	24
Rv2024c	8	21	31	41	76
Rv0124					31
Rv0286					30
Rv2770c					30
Rv1808					30
Rv3873	74	27	17	21	48
Rv2823c					27
Rv0690c					25
Rv2490c					23
Rv1789					22
Rv3135		3			40
Rv2123	77	16	1	2	36
Rv2892c					15
Rv1705c					12
Rv1387					12
Rv1802	51	9	10	31	11
Rv0293c					11
Rv2608					10
Rv3015c	10	3			26
Rv3021c					8
Rv3020c	1	56	5	7	46
Rv3136					6
Rv1243c	2	6			56
Rv3024c					2

Rank: 0 20 40 60 80 100
